# Natural Product Rottlerin Derivatives Targeting Quorum Sensing

**DOI:** 10.3390/molecules26123745

**Published:** 2021-06-19

**Authors:** Dittu Suresh, Shekh Sabir, Tsz Tin Yu, Daniel Wenholz, Theerthankar Das, David StC. Black, Naresh Kumar

**Affiliations:** 1 School of Chemistry, The University of New South Wales, Sydney, NSW 2052, Australia; d.suresh@student.unsw.edu.au (D.S.); s.sabir@student.unsw.edu.au (S.S.); tsztin.yu@unsw.edu.au (T.T.Y.); d.wenholz@unsw.edu.au (D.W.); 2 Department of Infectious Diseases and Immunology, School of Medical Sciences, The University of Sydney, Sydney, NSW 2006, Australia; das.ashishkumar@sydney.edu.au

**Keywords:** natural products, rottlerin, pyranochromene chalcone, quorum sensing inhibitors, *Pseudomonas aeruginosa*

## Abstract

Rottlerin is a natural product consisting of chalcone and flavonoid scaffolds, both of which have previously shown quorum sensing (QS) inhibition in various bacteria. Therefore, the unique rottlerin scaffold highlights great potential in inhibiting the QS system of *Pseudomonas aeruginosa.* Rottlerin analogues were synthesised by modifications at its chalcone- and methylene-bridged acetophenone moieties. The synthesis of analogues was achieved using an established five-step synthetic strategy for chalcone derivatives and utilising the Mannich reaction at C6 of the chromene to construct morpholine analogues. Several pyranochromene chalcone derivatives were also generated using aldol conditions. All the synthetic rottlerin derivatives were screened for QS inhibition and growth inhibition against the related LasR QS system. The pyranochromene chalcone structures displayed high QS inhibitory activity with the most potent compounds, **8b** and **8d**, achieving QS inhibition of 49.4% and 40.6% and no effect on bacterial growth inhibition at 31 µM, respectively. Both compounds also displayed moderate biofilm inhibitory activity and reduced the production of pyocyanin.

## 1. Introduction

Bacteria develop resistance due to the selective survival pressure applied by the mechanism of action of conventional antibiotics. Gram-negative bacteria are more resistant to antibiotics through some evolved mechanisms such as efflux pumps that eliminate the drugs, forming barriers that are more resistant to drug penetration, or producing enzymes that degrade drugs or through the formation of biofilm [1,2,3]. To overcome these resistance mechanisms, the concepts of bacterial biofilm formation and quorum sensing (QS) have attracted recent interest. As planktonic bacteria grow, small molecules known as autoinducers (AIs) are also synthesised intracellularly and released into the extracellular matrix. When the concentration of these AIs reaches a certain threshold, the bacteria reach a ‘quorum’, and the AIs bind to cognate receptors causing a signal transduction cascade that controls colony-wide gene expression. This type of intercellular bacterial communication, known as QS, is used to coordinate group behaviour and synchronise gene expression, including those responsible for biofilm formation as well as virulence [4,5]. 

**Figure 1:**
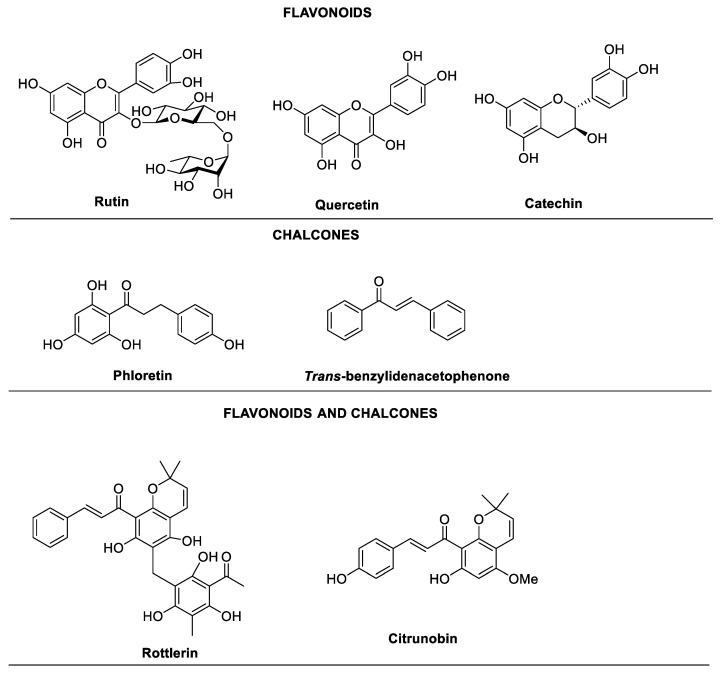
Flavonoid- and chalcone-containing natural products as anti-virulence and quorum sensing inhibitors.

Many natural products and their analogues have been tested for their potential as antibacterial agents. In particular, structures containing chalcones and flavonoids have stood out for their effectiveness as QS and biofilm inhibitors (Figure 1) [6]. Many traditional medicines and foods showing unique antibacterial properties have seen flavonoids as a recurring structural theme; one such naturally occurring flavonoid is quercetin. Quercetin has been reported to show activity against planktonic bacteria, as well as biofilm inhibition in both *Streptococcus pneumoniae* and *P. aeruginosa* [7,8]. Rutin and catechin are other examples of naturally occurring flavonoids that have been examined for QS activity. Rutin was found to significantly alter QS in *Escherichia coli,* inhibiting both the formation of biofilms and virulence genes [9], while catechin caused drastic negative effects in *P. aeruginosa* through the reduction of QS regulatory genes production (*lasI*, *lasR*, r*hlI*, and *rhlR)* as well as the inhibition of pyocyanin and biofilm formation [10]. Similarly, several natural chalcone structures have shown great potential in QS inhibition such as *trans*-benzylideneacetophenone (*trans*-chalcone) and licochalcone A. The *trans*-chalcone inhibited *Streptococcus mutans* biofilm formation while licochalcone showed an effective decrease in the expression of quorum-sensing genes in *Salmonella typhimurium* [11,12].

Rottlerin is a natural compound isolated from kamala, a red powder that is produced on the surface of the fruit of the endangered medicinal plant *Mallotus phillipinesis* [13]. Rottlerin, which possesses both the characteristics of a chalcone and a flavonoid, is a potent protein kinase C inhibitor with diverse biological activities. However, the limited availability of the natural molecule has resulted in restrictions to its development as a potential therapeutic lead. Recently, our group has reported an improved five-step large-scale synthesis of rottlerin [14] that can be completed in a time-efficient manner, thus creating an excellent opportunity to investigate its potential as a QS and biofilm inhibitor against Gram-negative bacteria, through the synthesis of analogues and developing structure–activity relationships. Moreover, we hypothesise that rottlerin analogues may target the quorum sensing systems in *P. aeruginosa***,** because various other natural product derivatives with flavonoid and chalcone structures have shown QS and biofilm inhibition marking potential for rottlerin to be a QS inhibitor [6,15]. 

In line with our continuous efforts to develop novel synthetic and natural product analogues as antibacterial agents [16,17,18], in this paper, we report the synthesis of biologically interesting analogues of rottlerin and pyranochromene chalcones. The synthesised compounds have been evaluated for quorum sensing, biofilm and pyocyanin inhibition in *P. aeruginosa*.

## 2. Results and Discussion

### 2.1. Analogue Synthesis 

**Scheme 1:**
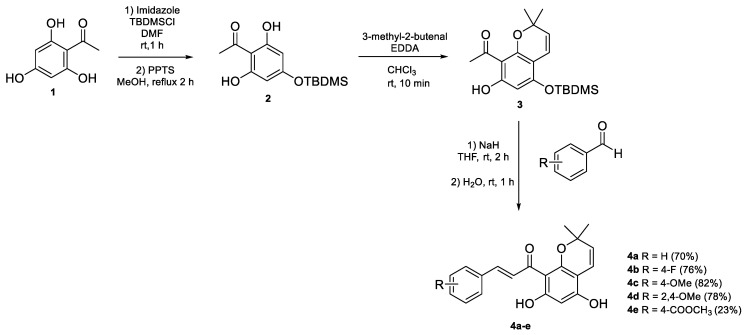
Synthesis of chromene chalcones derivatives **4a**–**e**.

The synthesis of rottlerin analogues followed the procedure previously described for the synthesis of rottlerin (Scheme 1) [14,19]. The synthesis involved the preparation of TBDMS-protected chromene **3** from 2,4,6-trihydroxyacetophenone **1** via mono TBDMS-protected acetophenone **2** followed by cyclisation with 3-methyl-2-butenal. Subsequently, chromene **3** underwent an aldol condensation reaction with benzaldehyde, resulting in the formation of chromene-chalcone **4**.

### 2.2. Mannich Reactions at the C6 Chromene Position

**Scheme 2:**
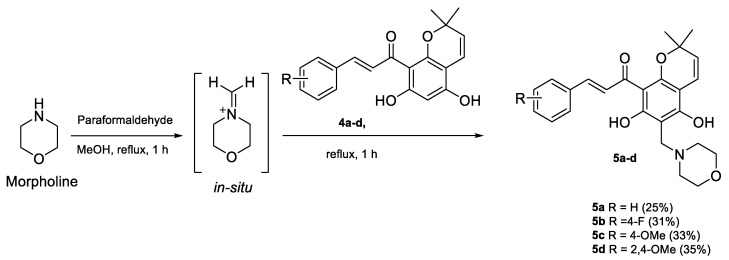
Synthesis of morpholine derivatives of chromene chalcones **5a**–**d**.

To understand the importance of the acetophenone group in rottlerin as well as to expand our library of compounds via Mannich reactions, the acetophenone group was replaced with a heterocyclic ring to produce a morpholine series of compounds **5a**–**d** (Scheme 2). These compounds were synthesised in low to moderate yields via the Mannich reaction using morpholine and paraformaldehyde to form an iminium ion that is then attacked by the electron rich aromatic ring of the chromene to form the methylene-bridged morpholine analogues. The ^1^H NMR spectra revealed the presence of a broad singlet (overlapping signals) at 3.68 to 3.94 ppm integrating for six protons, corresponding to the methylene bridge and morpholine ring N-(CH_2_)_2_ protons as well as a broad singlet at 2.70 to 2.80 ppm integrating for four protons representing the other two CH_2_ groups of the morpholine ring.

**Scheme 3:**
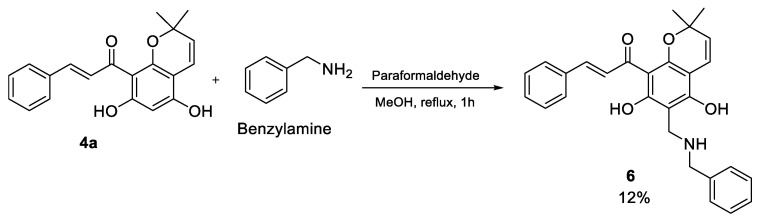
Synthesis of benzylamine analogue of chromene chalcones **6**.

Following the successful synthesis of the morpholine analogues, several amino acids including tryptamine, phenylalanine and proline were trialed using the same reaction conditions. However, these reactions were unsuccessful and generated various complex mixtures in which the desired product could not be detected. It was believed that this may be due to the zwitterionic nature of amino acids, making their amine group less reactive. Hence, the reaction was repeated using phenylalanine methyl ester. However, a similar result was observed, and this suggested that it is the carboxylate/ester group that may be interfering with the reaction. Evidence for this was provided by a subsequent reaction of benzylamine with **4a** under Mannich conditions that successfully yielded the final compound **6** in 12% isolated yield (Scheme 3). In the ^1^H NMR spectrum of compound **6** (Figure S15), the two methylene bridges neighbouring the NH were observed at 3.85 ppm and 4.12 ppm, while the five additional protons of the benzylamine ring were also consistent with the integration of the aromatic protons. Finally, HRMS analysis showed a *m*/*z* peak at 442.2013 corresponding to C_28_H_27_NO_4_ (required 442.2013), which matched the structure of the benzylamino derivative **6**.

### 2.3. Synthesis of Pyranochromenes

**Scheme 4:**
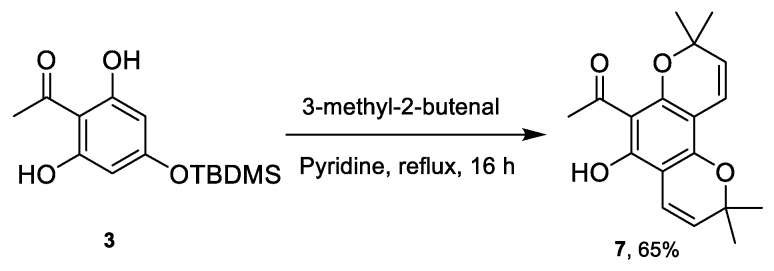
Synthesis of pyranochromene **7**.

Surprisingly, the synthesis of TBDMS-protected chromene 3 using pyridine as a base and a solvent instead of EDDA under reflux conditions resulted in the formation of an unexpected pyranochromene **7** (Scheme 4). It is believed that the high temperature reflux conditions caused a cleavage of the TBDMS-protecting group, resulting in the deprotected OH which further underwent cyclisation with 3-methyl-2-butenal to yield pyranochromene **7**. 

The ^1^H NMR spectrum of bicyclic chromene **7** showed the absence of the TBDMS group and the aromatic CH peak, but the appearance of an extra singlet at 1.24 ppm with an integration of six protons corresponding to the second dimethyl group. The spectrum also showed additional chromene CH=CH peaks which merged together at around 5.07 to 5.27 ppm and two doublets at 6.32 ppm and 6.39 ppm. In comparison, the analysis of the ^1^H NMR spectrum of TBDMS-protected chromene 3 clearly shows the *tert*-butyl group resonating as a singlet at 1.01 ppm with an integration of nine protons and the silyl-dimethyl group resonating at 0.26 ppm for six protons. Moreover, the presence of the aromatic CH peak at 5.96 ppm is also observed. 

### 2.4. Synthesis of Pyranochromene Chalcones ***8a***–***d***

**Scheme 5:**
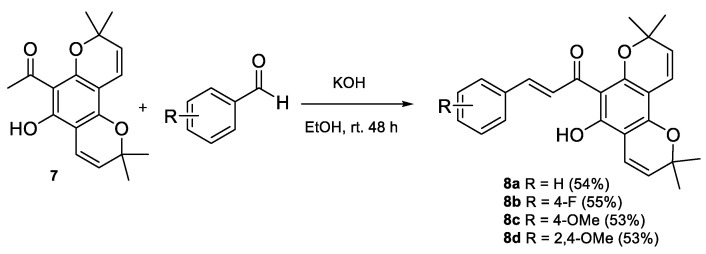
Synthesis of pyranochromene chalcones **8a**–**d**.

Following the synthesis of the pyranochromene compound, it was used to form chalcone derivatives. As there was no TBDMS-protecting group which needed cleavage under NaH conditions, compounds **8a**–**d** could be synthesised in moderate yields of 53 to 55% from pyranochromene **7** using the milder reaction condition (KOH in EtOH) and various substituted benzaldehydes with stirring at room temperature for 48 h (Scheme 5).

### 2.5. QSI and Growth Inhibition Results

The QSI activities of the synthesised compounds were evaluated against the LasR receptor by using the based MH602 P*_las_*B::*gfp* reporter strain of *P. aeruginosa* [20]. A known compound, furanone C-30 (Fu C-30), was used as a positive control [21]. 

The concentration-dependent QSI activities of the synthesised compounds against the LasR receptor of *P. aeruginosa* are shown in Table 1. Amongst all the tested compounds, the two pyranochromene compounds **8a**,**d** exhibited a promising QSI of 65.7% and 69.3%, respectively, at 125 µM, and 49.4% and 40.6%, respectively, at 31 µM. These compounds only possessed minimal (<5%) bacterial growth inhibition at all tested concentrations. 

Among three series (morpholine derivatives **5a**–**d**, pyranochromene derivatives **8a**–**d** and benzylamino derivative **6**) of compounds, pyranochromene derivatives **8a**–**d** exhibited superior QSI activities (59.5 to 69.3%) with minimal (<15%) bacterial growth inhibition at 125 µM. In terms of substitution at the terminal chalcone phenyl ring, the introduction of an electron-donating methoxy group or an electron-withdrawing fluorine atom had no significant effect on QSI activity at 125 µM. However, at the lower concentration of 31 µM, the fluoro-substituted analogue **8b** displayed a slightly higher QSI activity of 49.4% than its parent compound **8a** (QSI = 39.4%) and the methoxy-substituted analogues **8c**–**d** (QSI = 35.7% and 40.6%, respectively). All pyranochromene derivatives **8a**–**d** also showed 0% growth inhibitory activity at 31 µM concentration.

The morpholine compounds were more sensitive than the pyranochromene compounds to changes in the QSI activities as a result of chalcone modification. In terms of substitution at the terminal chalcone phenyl ring, the dimethoxy-substituted morpholine-bearing analogue **5d** possesses the highest QSI activity of 64.0% at 125 µM, and at a lower concentration of 31 µM, **5d** possesses a QSI activity of 42.6%. While QSI activities were expected to be concentration-dependent for all compounds, the opposite trend was observed for the mono-methoxy-substituted analogue **5c** for which the QSI activity increases from 29.1% at 125 µM to 57.9% at 31 µM. The reason of this is unclear and will require further investigation. Furthermore, when the morpholine moiety on compound **5a** was replaced by a benzylamino group as in compound **6**, the QSI activity increased from 28.0% to 40.8% at 125 µM, suggesting that the benzylamino group is favoured for QSI activity over the morpholine moiety at the high concentration of 125 µM. However, at the lower concentration of 31 µM, this relationship is less clear as compound **5a** (QSI = 30.0%) showed similar QSI activity to that of compound **6** (QSI = 24.6%). 

### 2.6. Pyocyanin and Biofilm Inhibition Results

**Figure 2:**
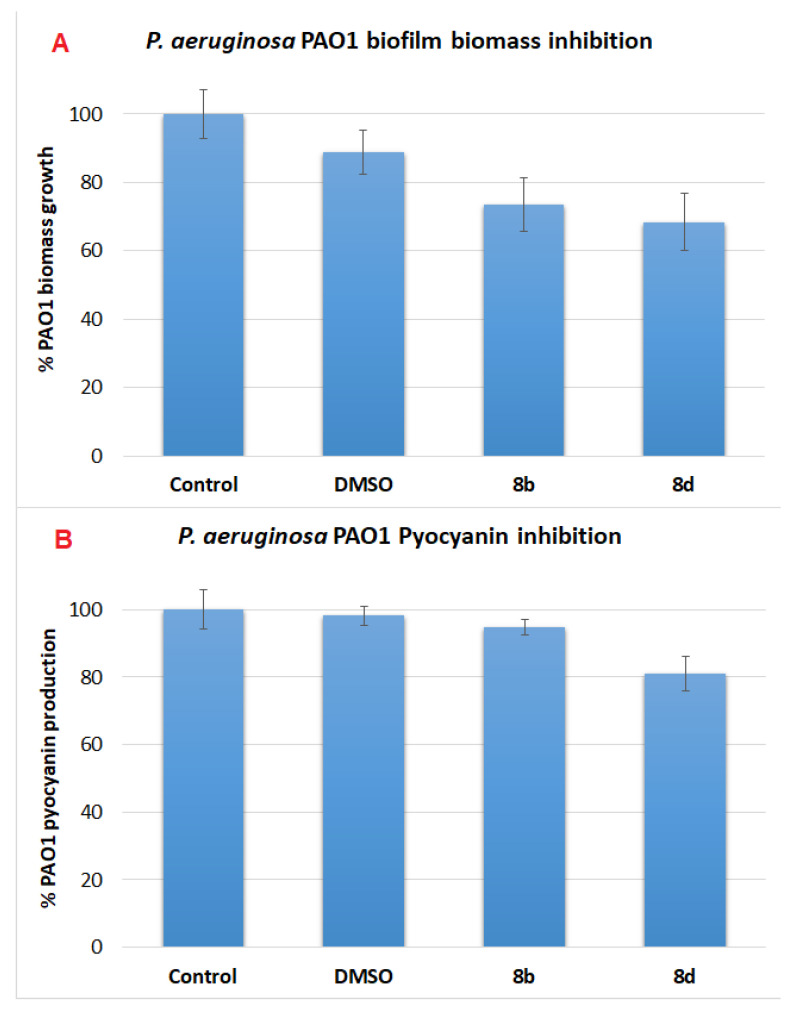
(**A**) Percentage biofilm mass inhibited by **8b**,**d** with blank as control and DMSO as comparison with *P. aeruginosa* (PAO1) planktonic cultures. (**B**) Percentage pyocyanin inhibited by **8b** and **8d** with respect to the DMSO control using *P. aeruginosa* (PAO1) planktonic cultures.

The more potent compounds, **8b**,**d**, which possess high QSI activities and low bacterial growth inhibition, were then selected to investigate their ability to inhibit pyocyanin production and biofilm formation (Figure 2). Both compounds only possess low to moderate antibacterial activity in the inhibition of the production of pyocyanin or formation of biofilm. Compound **8d** was more potent than compound **8b** with the percentage of pyocyanin inhibition and percentage inhibition of the formation of biofilm being 19% and 32%, respectively. This is interesting as compound **8b** was expected to possess a higher inhibitory activity in terms of pyocyanin production and biofilm formation because of its higher QSI activity. As other QS systems also contribute to pyocyanin production and biofilm formation, these results suggest that compound **8d** probably affects other QS systems, causing a higher overall inhibitory activity compared to compound **8b**. 

As QSI analogues derived from a natural product, the pyranochromene molecules **8b**,**d** in particular show great potential as they maintain above 40% inhibition of LasR QS at 31 µM concentration. There is a considerable amount of LasR QS inhibitors derived from natural products. Several flavanoid molecules with similar structural characteristics to the rottlerin analogues (naringenin, eriodictyol and taxifolin) from the plant Combretum albiflorum have previously been reported for QS activity and only naringenin showed significant LasR QS inhibition; however, this was at a significantly higher (4 mM) concentration in comparison [22]. Furthermore, *N*-decanoyl-l-homoserine benzyl ester and (phenylsulfinyl)alanine are other natural product derivatives which showed promise as QSI for LasR, with 29.67% inhibition at 100 µM and up to 87.2% inhibition at 1 mM respectively [23,24]. From these molecules, it is evident that the scaffolds for the pyranochromenes **8b**,**d** should be investigated further for their ability as QSIs at such a low concentration with no growth inhibition. Though there are also several other molecules which have been published that show greater effects, the pyranochromene rottlerine derivatives have the potential to be investigated further and be improved upon to the same levels.

## 3. Materials and Methods

### 3.1. Chemistry

All reagents were bought commercially from Sigma Aldrich (Castle Hill, NSW, Australia), Alfa Aesar (Haverhill, MA, USA) and Combi-Blocks (San Diego, California, United States) and used without further purification. Anhydrous solvents were acquired from PureSolv MD Solvent Purification System. Room temperature (rt) refers to the ambient temperature (25 °C). Reactions were monitored by thin-layer chromatography (TLC, Merck PTY Ltd., Bayswater, Victoria, Australia) using precoated Merck silica gel 60 F254 plates and visualised using UV light (254 nm). Bruker Avance III 300 (Bruker Pty Ltd., Preston, Victoria, Australia) and Bruker Avance III HD 400 (Bruker Pty Ltd., Preston, Victoria, Australia) were used to obtain all ^1^H and ^13^C NMR spectra with the respective solvents using chemical shifts (δ) in parts per million (ppm). Multiplicities for NMR spectra have been assigned using singlet (s), doublet (d), doublet of doublet (dd), doublet of doublet of doublet (ddd), doublet of triplet (dt), triplet (t), quartet (q), doublet of quartet (dq), pentet (p), hextet (h), septet (sept), multiplet (m), broad singlet (br) as necessary and coupling constants (*J*) in Hertz (Hz). Optimelt melting point apparatus was used for all measurement of all melting points, uncorrected. High-resolution mass spectra (HRMS, Thermo Scientific, Scoresby, Victoria, Australia) was conducted using Thermo LTQ Orbitrap XL instrument (Thermo Scientific, Scoresby, Victoria, Australia) under positive mode electrospray ionization by the UNSW Bioanalytical Mass Spectrometry Facility. Infrared (IR) spectra were recorded using a Cary 630 FTIR spectrometer (Agilent, Mulgrave, Victoria, Australia) fitted with a diamond-attenuated total reflectance (ATR) sample interface. Flash column chromatography was carried out using Grace Davisil LC60A silica. 

### 3.2. General Synthetic Procedure A for (E)-Chalcone Chromene Derivatives (***4a***–***e***)

TBDMS-protected chromene (**3**, 1.0 equivalent) was dissolved in anhydrous THF (10 mL) under an argon atmosphere and cooled to 0 °C. A measure of 60% NaH in mineral oil (5.0 equivalents) was added in small portions to the cooled solution over 5 min. The mixture was then stirred for 5 min before the addition of the appropriate substituted benzaldehyde (two equivalents) and stirring continued at room temperature for 2 h. Water (12 mL) was then added, and the reaction mixture was stirred for an additional 30 min. After completion of the reaction, the product was extracted into EtOAc (3 × 30 mL), combined organic extracts washed with brine (30 mL), dried over anhydrous sodium sulfate, and concentrated in vacuo. The crude mixture was purified using flash column chromatography on silica gel with *n*-hexane/EtOAc as the eluent to afford the product.

### 3.3. General Synthetic Procedure B for Mannich Reactions at C6 Chromene Position (***5a***–***d***, ***6***)

A solution of the appropriate amine or amino acid (1.2 equivalents) and paraformaldehyde (1.2 equivalents) were mixed in methanol and heated at reflux for 1 h. The relevant (*E*)-chalcone chromene derivative (**4a**–**e**, 1.0 equivalent) was added and the reaction mixture was heated at reflux for a further 2 h. After completing the reaction, the solvent was evaporated in vacuo and the resulting crude compound was purified using flash column chromatography on silica gel with DCM/MeOH as the eluent to afford the product.

### 3.4. Synthetic Procedure for Pyranochromene (***7***) 

3-Methyl-2-butenal (2.6 mL, 25.60 mmol) was added to a solution of 2 (2.89 g, 10.20 mmol) in pyridine (10 mL) and the reaction mixture was heated at reflux for 16 h. After completion of the reaction, the product was extracted into EtOAc (3 × 30 mL), the combined organic extracts were washed with brine (30 mL), dried over anhydrous sodium sulfate, and concentrated in vacuo. The crude mixture was purified using flash column chromatography on silica gel with *n*-hexane/EtOAc (19:1) as the eluent to afford the product as a yellow crystalline solid (2.00 g, 65%). mp 91.3 to 94.6 °C; ^1^H NMR (400 MHz, CDCl_3_) δ 14.00 (s, 1H), 6.65 (d, *J* = 10.0 Hz, 1H), 6.58 (d, *J* = 10.0 Hz, 1H), 5.47 to 5.42 (m, 2H), 2.66 (s, 3H), 1.50 (s, 6H), 1.44 (s, 6H); 13C NMR (101 MHz, CDCl_3_) δ 203.5, 160.7, 156.8, 155.2, 125.5, 124.9, 116.6, 116.3, 105.7, 102.5, 102.3, 33.4, 28.5, 28.1; IR (ATR): υ_max_ 3042, 2970, 2703, 1592 cm^−1^.

### 3.5. General Synthetic Procedure C for Pyranochromene Chalcones (***8a***–***d***)

The appropriate substituted benzaldehyde (1.2 equivalents) and KOH pellets (5.0 equivalents) were added to a solution of pyranochromene (**7**, 1.0 equivalent) in EtOH (10 mL). The reaction mixture was stirred at room temperature for 48 h, and following the completion of the reaction, EtOH was evaporated in vacuo. Water (10 mL) was then added to the crude product and extracted using EtOAc (3 × 20 mL). The combined organic extracts were washed with 2M HCl (15 mL) then brine (15 mL), dried over anhydrous sodium sulfate and concentrated in vacuo. The resulting product was purified using flash column chromatography on silica gel with *n*-hexane/EtOAc as the eluent to afford the product.

### 3.6. Experimental Characterisation Data

(*E*)-1-(5,7-Dihydroxy-2,2-dimethyl-2*H*-chromen-8-yl)-3-(4-fluorophenyl)prop-2-en-1-one (**4b**): The title compound was synthesised from TBDMS-protected chromene 3 (0.1 g, 0.287 mmol) and 4-fluorobenzaldehyde (0.061 mL, 0.574 mmol) following general synthetic procedure A. The product 4b was eluted and obtained as an orange solid (0.74g, 0.218mmol); mp 169.6 to 174.7 °C; ^1^H NMR (400 MHz, CDCl_3_) δ 13.97 (s, 1H), 8.01 (d, *J* = 15.6 Hz, 1H), 7.73 (d, *J* = 15.6 Hz, 1H), 7.62 to 7.54 (m, 2H), 7.15 to 7.06 (m, 2H), 6.57 (d, *J* = 9.9 Hz, 1H), 5.95 (s, 1H), 5.50 (d, *J* = 9.9 Hz, 1H), 5.46 (s, 1H), 1.55 (s, 6H); ^13^C NMR (101 MHz, CDCl3) δ 192.91, 166.72, 157.84, 156.82, 141.21, 131.97, 130.24, 130.15, 127.33, 125.07, 116.50, 116.40, 116.18, 106.85, 102.37, 96.57, 78.40, 28.19; IR (ATR): υ_max_ 3238, 2967, 2342, 2120, 1591, 1504 cm**^−^**^1^; MS (+ESI): *m/z* 341.1184, [M + H]^+^, C_20_H_17_FO_4_ required [M + H]^+^ 341.1184.

(*E*)-1-(5,7-Dihydroxy-2,2-dimethyl-2*H*-chromen-8-yl)-3-(4-methoxyphenyl)prop-2-en-1-one (**4c**): The title compound was synthesised from TBDMS-protected chromene 3 (0.5 g, 1.435 mmol) and 4-methoxybenzaldehyde (0.35 mL, 2.87 mmol) following general synthetic procedure A. The product 4c was obtained as an orange solid (0.415 g, 82%); mp 193.3–196.2 °C; ^1^H NMR (400 MHz, CDCl_3_) δ 14.15 (s, 1H), 8.00 (d, *J* = 15.6 Hz, 1H), 7.77 (d, *J* = 15.6 Hz, 1H), 7.60 to 7.52 (m, 2H), 6.98 to 6.87 (m, 2H), 6.57 (d, *J* = 9.9 Hz, 1H), 5.94 (s, 1H), 5.49 (d, *J* = 9.9 Hz, 1H), 3.86 (s, 3H), 1.56 (s, 6H); IR (ATR): υ_max_ 3178, 3122, 2964, 2942, 2321, 2116, 1588 cm**^−^**^1^; MS (+ESI): *m/z* 353.1383, [M + H]^+^, C_21_H_20_O_5_ required [M + H]^+^ 353.1384.

(*E*)-1-(5,7-Dihydroxy-2,2-dimethyl-2*H*-chromen-8-yl)-3-(2,4-dimethoxyphenyl)prop-2-en-1-one (**4d**): The title compound was synthesised from TBDMS-protected chromene 3 (0.4 g, 1.148 mmol) and 2,4-dimethoxybenzaldehyde (0.38 g, 2.296 mmol) following general synthetic procedure A. The product 4d was obtained as a dark orange solid (0.342 mg, 78%); mp 162.8 to 163.7 °C; ^1^H NMR (300 MHz, CDCl_3_) δ 14.27 (s, 1H), 8.13 (d, *J* = 15.7 Hz, 1H), 8.02 (d, *J* = 15.7 Hz, 1H), 7.56 (d, *J* = 8.6 Hz, 1H), 6.60 to 6.46 (m, 3H), 5.94 (s, 1H), 5.48 (d, *J* = 9.9 Hz, 1H), 3.89 (s, 3H), 3.86 (s, 3H), 1.54 (s, 6H); ^13^C NMR (101 MHz, CDCl3) δ 193.50, 166.65, 163.03, 160.38, 157.61, 156.72, 138.30, 129.97, 125.26, 124.98, 117.92, 116.63, 106.95, 105.68, 102.33, 98.62, 96.50, 78.14, 62.05, 55.74, 55.64, 28.04^.^; IR (ATR): υ_max_ 3199, 2962, 2931, 2168, 1602 cm**^−^**^1^; MS (+ESI): *m/z* 383.1490, [M + H]^+^, C_22_H_22_O_6_ required [M + H]^+^ 383.1489.

Methyl(*E*)-4-(3-(5,7-dihydroxy-2,2-dimethyl-2*H*-chromen-8-yl)-3-oxoprop-1-en-1-yl) benzoate (**4e**): The title compound was synthesised from TBDMS-protected chromene 3 (0.1 g, 0.327 mmol) and methy-4-formylbenzoate (0.140 g, 0.854 mmol) following general synthetic procedure A. The product 4e was obtained as a yellow solid (0.028 g, 23%); mp 211.3–215.6 °C; ^1^H NMR (400 MHz, DMSO-*d_6_*) δ 13.86 (s, 1H), 8.14 (d, *J* = 15.6 Hz, 1H), 8.10 to 8.04 (m, 2H), 7.74 (d, *J* = 15.6 Hz, 1H), 7.70 to 7.62 (m, 2H), 6.57 (d, *J* = 9.9 Hz, 1H), 5.95 (s, 1H), 5.50 (d, *J* = 9.9 Hz, 1H), 3.94 (s, 3H), 1.55 (s, 6H); IR (ATR): υ_max_ 3198, 2973, 2706, 2304, 2107, 1931, 1719, 1601 cm**^−^**^1^; MS (+ESI): *m/z* 381.1331, [M + H]^+^, C_22_H_20_O_6_ required [M + H]^+^ 381.1333.

(*E*)-1-(5,7-Dihydroxy-2,2-dimethyl-6-(morpholinomethyl)-2*H*-chromen-8-yl)-3-phenylprop-2-en-1-one (**5a**): The title compound was synthesised from **4a** (0.1 g, 0.31 mmol), paraformaldehyde (0.011 g, 0.37 mmol) and morpholine (0.032 mL, 0.37 mmol) following general synthetic procedure B. The product 7a was obtained as an orange solid (0.033 g, 25%); mp 131.2 to 134.5 °C; ^1^H NMR (400 MHz, CDCl_3_) δ 8.13 (d, *J* = 15.7 Hz, 1H), 7.76 (d, *J* = 15.7 Hz, 1H), 7.64 to 7.57 (m, 2H), 7.46 to 7.34 (m, 3H), 6.63 (d, *J* = 9.9 Hz, 1H), 5.47 (d, *J* = 9.9 Hz, 1H), 3.91 to 3.68 (m, 6H), 2.78 (br, 4H), 1.55 (s, 6H); ^13^C NMR (76 MHz, DMSO) δ 190.78, 164.59, 164.06, 155.24, 141.24, 135.16, 130.41, 129.30, 128.12, 127.34, 124.26, 116.82, 103.43, 102.68, 98.87, 77.84, 65.51, 53.05, 51.93, 27.62^.^; IR (ATR): υ_max_ 2957, 2846, 2521, 2343, 2118, 1848, 1599 cm**^−^**^1^; MS (+ESI): *m/z* 422.1961, [M + H]^+^. C_25_H_27_NO_5_ required [M + H]^+^ 422.1962.

(*E*)-1-(5,7-Dihydroxy-2,2-dimethyl-6-(morpholinomethyl)-2*H*-chromen-8-yl)-3-(4-fluorophenyl)prop-2-en-1-one (**5b**): The title compound was synthesised from 4b (0.2 g, 0.56 mmol), paraformaldehyde (0.020 g, 0.68 mmol) and morpholine (0.06 mL, 0.68 mmol) following general synthetic procedure B. The product 5b was obtained as an orange solid (0.068 g, 31%); mp 148.2 to 152.1 °C; ^1^H NMR (400 MHz, CDCl_3_) δ 8.04 (d, *J* = 15.6 Hz, 1H), 7.72 (d, *J* = 15.6 Hz, 1H), 7.63 to 7.54 (m, 2H), 7.16 to 7.06 (m, 2H), 6.65 (d, *J* = 9.9 Hz, 1H), 5.47 (d, *J* = 9.9 Hz, 1H), 3.94 to 3.75 (m, 6H), 2.80 (br, 4H), 1.54 (s, 6H); ^13^C NMR (101 MHz, CDCl_3_) δ 192.73, 180.10, 165.03, 164.79, 162.69, 141.00, 132.05, 130.21, 130.12, 127.44, 124.62, 117.08, 116.39, 116.17, 105.40, 103.18, 99.02, 97.19, 78.36, 66.33, 52.62, 28.29; IR (ATR): υ_max_ 2922, 2854, 2501, 2343, 2113, 1810, 1596 cm**^−^**^1^; MS (+ESI): *m/z* 440.1865, [M + H]^+^. C_25_H_26_FNO_5_ required [M + H]^+^ 440.1868.

(*E*)-1-(5,7-Dihydroxy-2,2-dimethyl-6-(morpholinomethyl)-2*H*-chromen-8-yl)-3-(4-methoxyphenyl)prop-2-en-1-one (**5c**).The title compound was synthesised from **4c** (0.05 g, 0.14 mmol), paraformaldehyde (0.05 g, 0.17 mmol) and morpholine (0.018 mL, 0.17 mmol) following general synthetic procedure B. The product **7c** was obtained as an orange solid (0.02 g, 33%); mp 143.3 to 146.2 °C; ^1^H NMR (400 MHz, CDCl_3_) δ 8.03 (d, *J* = 15.6 Hz, 1H), 7.76 (d, *J* = 15.6 Hz, 1H), 7.60 to 7.50 (m, 2H), 6.98 to 6.90 (m, 2H), 6.64 (d, *J* = 9.9 Hz, 1H), 5.47 (d, *J* = 9.9 Hz, 1H), 4.04 to 3.69 (m, 9H), 2.70 (br, 4H), 1.55 (s, 6H); IR (ATR): υ_max_ 2962, 2915, 2852, 2556, 1600, 1542 cm**^−^**^1^; MS (+ESI): *m/z* 452.2065, [M + H]^+^, C_26_H_29_NO_6_ required [M + H]^+^ 452.2068.

(*E*)-1-(5,7-Dihydroxy-2,2-dimethyl-6-(morpholinomethyl)-2*H*-chromen-8-yl)-3-(2,4-dimethoxyphenyl)prop-2-en-1-one (**5d**). The title compound was synthesised from 4d (0.05 g, 0.13 mmol), paraformaldehyde (0.05 g, 0.17 mmol) and morpholine (0.018 mL, 0.17 mmol) following general synthetic procedure B. The product 7d was obtained as an orange solid (0.021 g, 35%); mp 139.5 to 142.7 °C; ^1^H NMR (400 MHz, DMSO) δ 8.04 to 7.88 (m, 2H), 7.58 (d, *J* = 8.3 Hz, 1H), 6.67 (d, *J* = 8.0 Hz, 2H), 6.53 (d, *J* = 9.9 Hz, 1H), 5.54 (d, *J* = 9.9 Hz, 1H), 3.89 (s, 3H), 3.84 (s, 3H), 3.79 (s, 2H), 3.71 to 3.62 (m, 4H), 2.68 to 2.58 (m, 4H), 1.49 (s, 6H); ^13^C NMR (101 MHz, DMSO-*d_6_*) δ 191.56, 163.71, 162.94, 162.80, 159.89, 154.95, 137.31, 129.76, 124.48, 124.41, 116.59, 116.35, 106.56, 103.78, 102.18, 98.97, 98.68, 77.67, 65.69, 55.93, 55.58, 53.17, 52.04, 27.44; IR (ATR): υ_max_ 2923, 2846, 2614, 2508, 2343, 2113, 1869, 1596 cm**^−^**^1^; MS (+ESI): *m/z* 452.2173, [M + H]^+^, C_27_H_31_NO_7_ required [M + H]^+^ 482.2173.

(*E*)-1-(6-((Benzylamino)methyl)-5,7-dihydroxy-2,2-dimethyl-2H-chromen-8-yl)-3-phenylprop-2-en-1-one (**6**): The title compound was synthesised from **4a** (0.05 g, 0.15 mmol), paraformaldehyde (0.006 g, 0.18 mmol) and benzylamine (0.019 g, 0.18 mmol) following the general synthetic procedure B. The product **8a** was obtained as a yellow solid (0.008 g); mp 132.4 to 135.2 °C; ^1^H NMR (300 MHz, CDCl_3_) δ 8.15 (d, *J* = 15.7 Hz, 1H), 7.75 (d, *J* = 15.7 Hz, 1H), 7.67 to 7.56 (m, 2H), 7.48 to 7.27 (m, 8H), 6.66 (d, *J* = 9.9 Hz, 1H), 5.46 (d, *J* = 9.9 Hz, 1H), 4.13 (s, 2H), 3.85 (s, 2H), 1.55 (s, 6H); IR (ATR): υ_max_ 3158, 2957, 2546, 2321, 2112, 1910, 1592 cm**^−^**^1^; MS (+ESI): *m/z* 442.2013, [M + H]^+^, C_28_H_27_NO_4_ required [M + H]^+^ 442.2013.

(*E*)-1-(5-Hydroxy-2,2,8,8-tetramethyl-2H,8H-pyrano [2,3-f]chromen-6-yl)-3-phenylprop-2-en-1-one (**8a**): The title compound was synthesised from **7** (0.05 g, 0.17 mmol), benzaldehyde (0.02 mL, 0.21 mmol) and KOH pellets (0.05 g, 0.85 mmol) following general synthetic procedure C. The product **8a** was obtained as an orange solid (0.036 g, 54%); mp 95.2–97.4 °C; ^1^H NMR (400 MHz, CDCl_3_) δ 14.36 (s, 1H), 8.10 (d, *J* = 15.6 Hz, 1H), 7.77 (d, *J* = 15.6 Hz, 1H), 7.64 to 7.57 (m, 2H), 7.46 to 7.34 (m, 3H), 6.69 (d, *J* = 10.0 Hz, 1H), 6.62 (d, *J* = 10.0 Hz, 1H), 5.52 to 5.45 (m, 2H), 1.55 (s, 6H), 1.46 (s, 6H); ^13^C NMR (101 MHz, CDCl_3_) δ 193.08, 164.25, 161.62, 156.37, 142.22, 135.82, 130.19, 129.11, 128.40, 127.77, 125.55, 124.93, 116.75, 116.40, 102.78, 78.47, 78.44, 54.67, 28.57, 28.25; IR (ATR): υ_max_ 3058, 2969, 2644, 2342, 2109, 1733, 1580 cm**^−^**^1^; MS (+ESI): *m/z* 389.1748, [M + H]^+^, C_25_H_24_O_4_ required [M + H]^+^ 389.1747.

(*E*)-3-(4-Fluorophenyl)-1-(5-hydroxy-2,2,8,8-tetramethyl-2H,8H-pyrano[2,3-f]chromen-6-yl)prop-2-en-1-one (**8b**):The title compound was synthesised from **7** (0.05 g, 0.17 mmol), 4-fluorobenzaldehyde (0.023 mL, 0.21 mmol) and KOH pellets (0.05 g, 0.85 mmol) following general synthetic procedure C. The product **8b** was obtained as an orange solid (0.38 g, 55%); mp 121.6 to 125.7 °C; ^1^H NMR (400 MHz, CDCl_3_) δ 14.31 (s, 1H), 8.01 (d, *J* = 15.6 Hz, 1H), 7.72 (d, *J* = 15.6 Hz, 1H), 7.63 to 7.53 (m, 2H), 7.15 to 7.06 (m, 2H), 6.69 (d, *J* = 10.0 Hz, 1H), 6.62 (d, *J* = 10.0 Hz, 1H), 5.52 to 5.40 (m, 2H), 1.54 (s, 6H), 1.46 (s, 6H); IR (ATR): **υ**_max_ 3056, 2969, 2343, 2116, 1883, 1584 cm**^−^**^1^; MS (+ESI): *m/z* 407.1655, [M + H]^+^, C_25_H_23_FO_4_ required [M + H]^+^ 407.1653.

(*E*)-1-(5-Hydroxy-2,2,8,8-tetramethyl-2*H*,8*H*-pyrano[2,3-f]chromen-6-yl)-3-(4-methoxyphenyl)prop-2-en-1-one (**8c**): The title compound was synthesised from **7** (0.05 g, 0.17 mmol), 4-methoxybenzaldehyde (0.025 mL, 0.21 mmol) and KOH pellets (0.05 g, 0.85 mmol) following general synthetic procedure C. The product **10c** was obtained as a red solid (0.38 g, 53%); mp 134.3 to 135.7; ^1^H NMR (400 MHz, CDCl_3_) δ 8.00 (d, *J* = 15.6 Hz, 1H), 7.76 (d, *J* = 15.6 Hz, 1H), 7.56 (d, *J* = 8.8 Hz, 2H), 6.94 (d, *J* = 8.8 Hz, 2H), 6.69 (d, *J* = 9.9 Hz, 1H), 6.62 (d, *J* = 9.9 Hz, 1H), 5.50 to 5.44 (m, 2H), 3.85 (s, 3H), 1.55 (s, 6H), 1.46 (s, 6H); ^13^C NMR (101 MHz, CDCl_3_) δ 193.02, 171.28, 161.65, 161.48, 156.27, 155.23, 142.33, 130.10, 128.56, 125.48, 125.40, 124.84, 116.81, 116.46, 114.59, 106.10, 102.78, 102.64, 78.37, 78.32, 55.54, 28.53, 28.24^.^; IR (ATR): υ_max_ 3084, 2967, 2931, 2513, 2322, 2116, 1814, 1576 cm**^−^**^1^; MS (+ESI): *m/z* 441.1668, [M + Na]^+^, C_26_H_26_O_5_ required [M + Na]^+^ 441.1672.

(*E*)-3-(2,4-Dimethoxyphenyl)-1-(5-hydroxy-2,2,8,8-tetramethyl-2H,8H-pyrano[2,3-f]chromen-6-yl)prop-2-en-1-one (**8d**): The title compound was synthesised from **7** (0.05 g, 0.17 mmol), 2,4-dimethoxybenzaldehyde (0.035 mg, 0.21 mmol) and KOH pellets (0.05 g, 0.85 mmol) following general synthetic procedure C. The product **8d** was obtained as a red solid (0.40 g, 53%); mp 133.5 to 138.4 °C; ^1^H NMR (300 MHz, CDCl_3_) δ 8.12 (d, *J* = 15.7 Hz, 1H), 8.02 (d, *J* = 15.7 Hz, 1H), 7.56 (d, *J* = 8.6 Hz, 1H), 6.70 (d, *J* = 10.0 Hz, 1H), 6.61 (d, *J* = 10.0 Hz, 1H), 6.57 to 6.51 (m, 1H), 6.49 to 6.45 (m, 1H), 5.51 to 5.42 (m, 2H), 5.30 (s, 1H), 3.88 (s, 3H), 3.86 (s, 3H), 1.53 (s, 7H), 1.45 (s, 6H); ^13^C NMR (76 MHz, CDCl_3_) δ 193.46, 162.96, 161.66, 160.32, 156.24, 154.99, 137.91, 129.89, 125.49, 125.38, 124.86, 117.98, 116.84, 116.57, 106.23, 105.64, 102.76, 102.57, 98.61, 78.25, 78.19, 55.73, 55.63, 53.57, 28.51, 28.11; IR (ATR): υ_max_ 3102, 2970, 2935, 2342, 2116, 1736, 1606 cm**^−^**^1^; MS (+ESI): *m/z* 449.1958, [M + H]^+^, C_27_H_28_O_6_ required [M + H]^+^ 449.1959.

### 3.7. Biological Assays

#### 3.7.1. LasR QS and Growth Inhibition Assay

The *P. aeruginosa* MH602 P*_las_*B::*gfp*(ASV) reporter strain, which harbors a chromosomal fusion of the *lasB* promoter to an unstable *gfp* gene and responds to its autoinducer OdDHL [25], was used to evaluate the QSI activity of the synthesised compounds on QS signalling. An overnight culture was prepared in Luria-Bertani (LB10) media supplemented with gentamycin (40 μM). This culture was then diluted (1:100) in TSB/LB10 (4:1) medium supplemented with gentamycin (40 μM) and 200 μL aliquots were dispensed to 96-well plate flat-bottom wells (Costar). The culture was supplemented with varying concentrations of test compounds dissolved in DMSO, with the final concentration of test compounds being 125, 62.5 and 31.25 μM. Wells with bacterial culture but no compound were used as negative controls while wells supplemented with furanone C-30 (Fu C-30) were used as positive controls. The plates were incubated for 15 h at 37 °C with shaking at 150 rpm. After incubation, the reading of fluorescence (excitation, 485 nm; emission, 520 nm) and cell growth (optical density (OD) at 600 nm) was taken in a plate reader (FLUOstar Omega, BMG Labtech). A decrease in fluorescence corresponded to increased QSI and lowered activation of the QS system.

#### 3.7.2. Pyocyanin and Biofilm Inhibition Assay

*P. aeruginosa* planktonic cultures of PAO1 were grown overnight at 37 °C and 150 rpm in *Tryptone soya* broth (TSB, Oxoid, Thermo Scientific, Scoresby, Victoria, Australia). A total of 200 µL (0.1 ± 0.02 at OD 600nm) of bacterial cell density was added into a 96-well plate (Corning Corp, Corning, NY, USA) with the compounds 8b and 8d (50 µM) with 48 h of incubation to initiate biofilm formation. The controls used blank and DMSO/solvent to initiate biofilm formation; after 48 h, PBS was used to wash the formed biofilms once, the 96-well plates were then stained with 200 µL 0.1% (*w*/*v*) crystal violet (CV) and incubated at 37 °C and 150 rpm for 1 h. This was followed by three washes of PBS removing excess CV, 15 min of drying at 37 °C and dissolving in 80% *v*/*v* ethanol, then transferring into a new 96-well, where the biomass was quantified at OD 550 nm. Using the blank as a control, the activity of compounds **8b** and **8d** were tested as a percentage decrease from the blank control and was also compared to the effect of DMSO by itself. 

For pyocyanin quantification, planktonic cultures of *P. aeruginosa* PAO1 were grown overnight by inoculating a single colony of PAO1 from the tryptone soy agar plate in 5 mL tryptone soy broth (TSB) and incubating at 37 °C and 100 rpm. After overnight growth, the bacterial cultures were further diluted in 5 mL of TSB to a OD_600nm_ = 0.2 ± 0.02. The diluted bacterial suspension was grown for 48 h (at 37 °C and 100 rpm) either in the presence or absence of DMSO and compounds (50 µM) 8b and **8d**. Afterwards, the planktonic cultures were transferred into sterile falcon tubes and centrifuged at 4500× *g* for 10 min at 4 °C. The PAO1 supernatant was then removed by pipetting and transferred into new falcon tubes, followed by the addition of chloroform to a dilution factor of 300 µL of chloroform for every 1000 µL of supernatant. The supernatant–chloroform mixture was vortexed for 5 s and centrifuged at 4500× *g* for 10 min at 4 °C. Chloroform separated the pyocyanin in the supernatant into a blue layer. The blue layer was carefully pipetted out into sterile falcon tubes and treated with 0.2 M hydrochloric acid, to a ratio of 1:2 (i.e., one part of 0.2 M HCl to two parts of blue colour solution). The HCl-blue pyocyanin mixture was then immediately vortexed for 5 s and further centrifuged (10 min, 4500× *g*: 4 °C). The resultant acidified pyocyanin appeared as a red/pink layer at the top, of which 200 uL was aliquoted into a 96-well plate, and the absorbance was recorded at OD_520nm_ using a plate reader. The pyocyanin extraction assay using chloroform-HCl was adopted from Essar et al. 1990 [26].

## 4. Conclusions

A library of nine rottlerin analogues was successfully designed and synthesised for targeting the QS system in *P. aeruginosa*. The Mannich reaction at the C6 of chromene was utilised for the generation of methylene-bridged morpholine analogues as well as benzylaminomethyl compound **6**. Furthermore, the unexpected synthesis of the pyranochromene led to the synthesis of four promising pyranochromene chalcone analogues. The synthesised compounds were tested with the LasR receptor; the pyranochromene structures exhibited good LasR QSI activity for all derivatives while also displaying low bacterial growth inhibition and the most potent analogues were identified to be **8b**,**d** with 49% and 40% QSI at 31 µM. These two compounds were also tested for their effect on biofilm and pyocyanin inhibition, with **8d** presenting promising results of 19% and 32%, respectively. Contrastingly, the morpholine analogues showed much lower activity, except for **5d,** which displayed undesirably high growth inhibition. Based on these results the chromene structure was identified to be crucial in maintaining good LasR-based QSI activity in conjunction with low bacterial growth inhibition in Gram-negative bacteria. Additionally, due to the effectiveness of the pyranochromene chalcones in LasR, analogues of these structures can be further developed towards examining SAR.

## Figures and Tables

**Table 1:** QSI inhibition assay on LasR system of *P. aeruginosa* using percentage of GFP fluorescence at 485 nm in P*_las_*B::*gfp*(ASV). All measurements were taken from at least two independent tests carried out in triplicates with ± standard deviation from mean.

Compound	125 µM	63 µM	31 µM
	Mean Percentage Inhibition		
**5a**	28.0 ± 4.5 ^b^	26.8 ± 6.7 ^a^	30.0 ± 2.7 ^a^
**5b**	40.3 ± 3.8 ^a^	27.4 ± 10.2 ^a^	13.8 ± 10.0 ^a^
**5c**	29.1 ± 16.7 ^b^	48.8 ± 13.3 ^b^	57.9 ± 3.6 ^b^
**5d**	64.0 ± 3.4 ^c^	47.9 ± 5.1 ^b^	42.6 ± 0.9 ^c^
**6**	40.8 ± 4.2 ^b^	24.3 ± 3.2 ^a^	24.6 ± 1.1 ^b^
**8a**	66.3 ± 6.9 ^a^	52.7 ± 5.8 ^a^	39.4 ± 3.3 ^a^
**8b**	65.7 ± 3.7 ^a^	58.1 ± 9.4 ^a^	49.4 ± 1.5 ^a^
**8c**	59.5 ± 0.6 ^a^	46.5 ± 6.1 ^a^	35.7 ± 3.3 ^a^
**8d**	69.3 ± 10.5 ^a^	58.4 ± 7.3 ^a^	40.6 ± 4.0 ^a^
**Fu30**	95.5 ± 1.7 ^c^	90.7 ± 3.4 ^c^	93.1 ± 1.9 ^b^

^a^ Growth inhibition less than 15%. ^b^ Growth inhibition >15% but <30%. ^c^ Growth inhibition greater than 30%.

## Data Availability

Data is contained within the article and Supplementary Materials.

## Supplementary Materials

The supplementary materials are available online. 1H and 13C NMR spectra of the compounds and Growth inhibition data (*P. aeruginosa* MH602).

## Sample Availability

Not available.
