# Supplementary material for: Natural Product Rottlerin Derivatives Targeting Quorum Sensing
Source: Molecules. 2021 Jun 19;26(12):3745. doi: 10.3390/molecules26123745 (PMC8235494; doi:10.3390/molecules26123745)
Supplement: Supplementary file 1 [file molecules-26-03745-s001.zip › molecules-1247669-SI.pdf]

Supporting information

Natural Product Rottlerin Derivatives Targeting Quorum Sensing

Dittu Suresh [1], Shekh Sabir [1], Tsz Tin Yu[1], Daniel Wenholz[1], Theerthankar Das[2], David StC. Black[1] and Naresh Kumar [1,*]

[1] School of Chemistry, The University of New South Wales, NSW 2052 Sydney, Australia; d.suresh@student.unsw.edu.au (D.S.); s.sabir@student.unsw.edu.au (S.S.); tsztin.yu@unsw.edu.au (T.T.Y.); d.wenholz@unsw.edu.au (D.W); d.black@unsw.edu.au (D.StC.B.).

[2] Department of Infectious Diseases and Immunology, School of Medical Sciences, The University of Sydney, NSW 2006 Sydney, Australia; das.ashishkumar@sydney.edu.au.

* Correspondence: n.kumar@unsw.edu.au; Tel.: +61-29385-4698; Fax: +61-29385-6141.

Contents

^{1}H and ^{13}C NMR spectra of the compounds S2-S24

Growth inhibition data (*P.aeruginosa* MH602)... …S25

^{1}H NMR spectrum of compound **3**

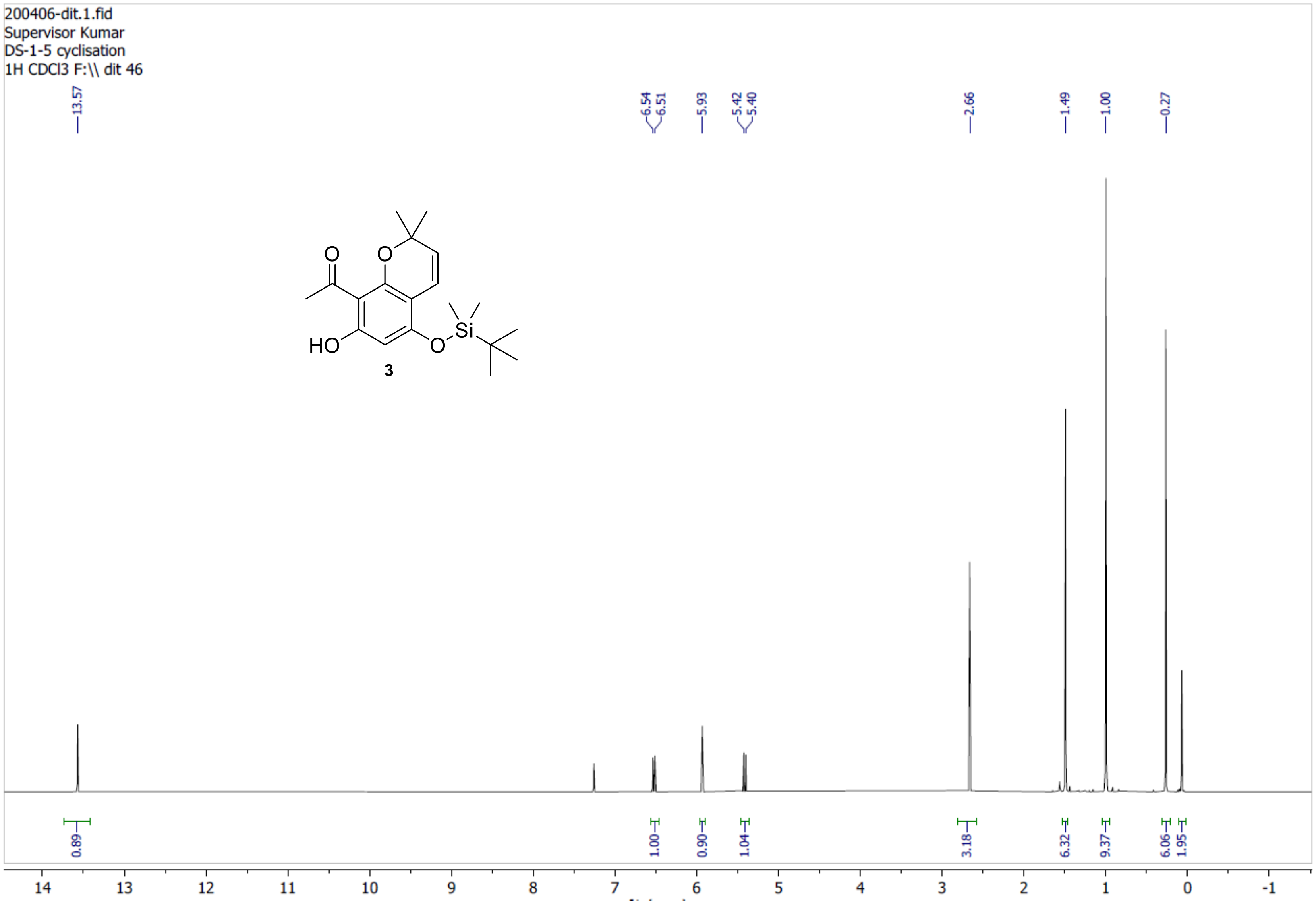

^{1}H NMR spectrum of compound **4b**

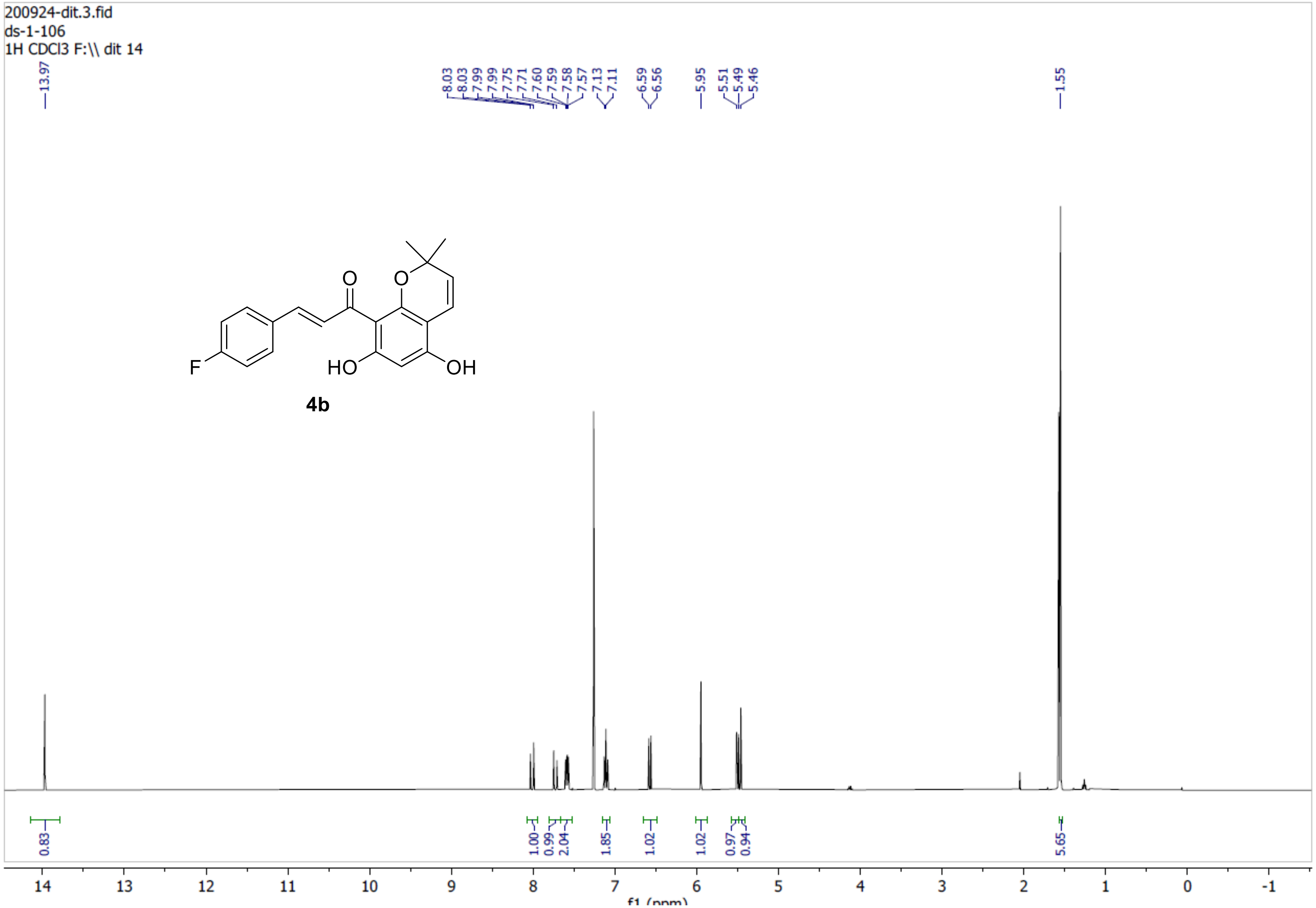

^{13}C NMR spectrum of compound **4b**

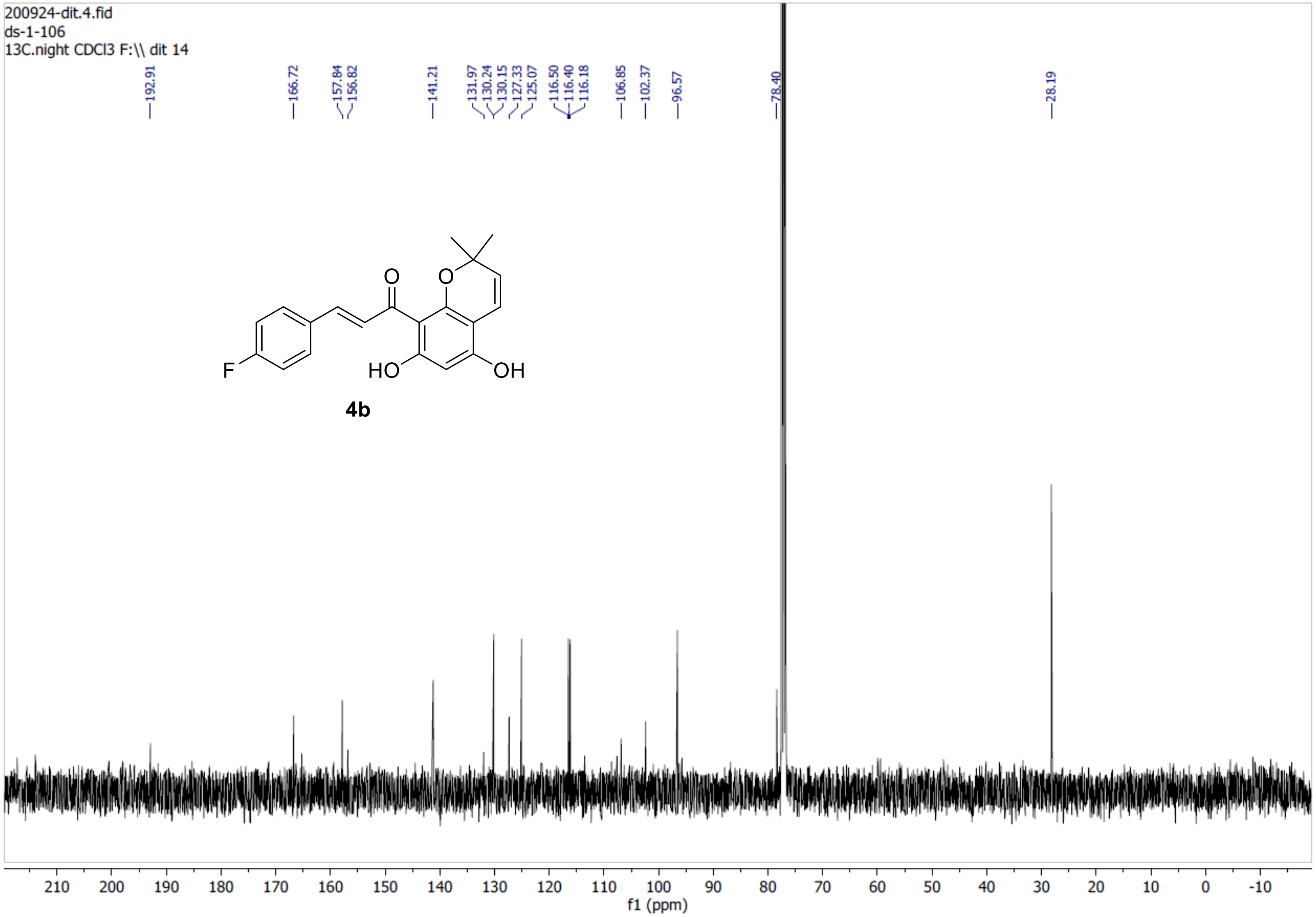

^{1}H NMR spectrum of compound **4c**

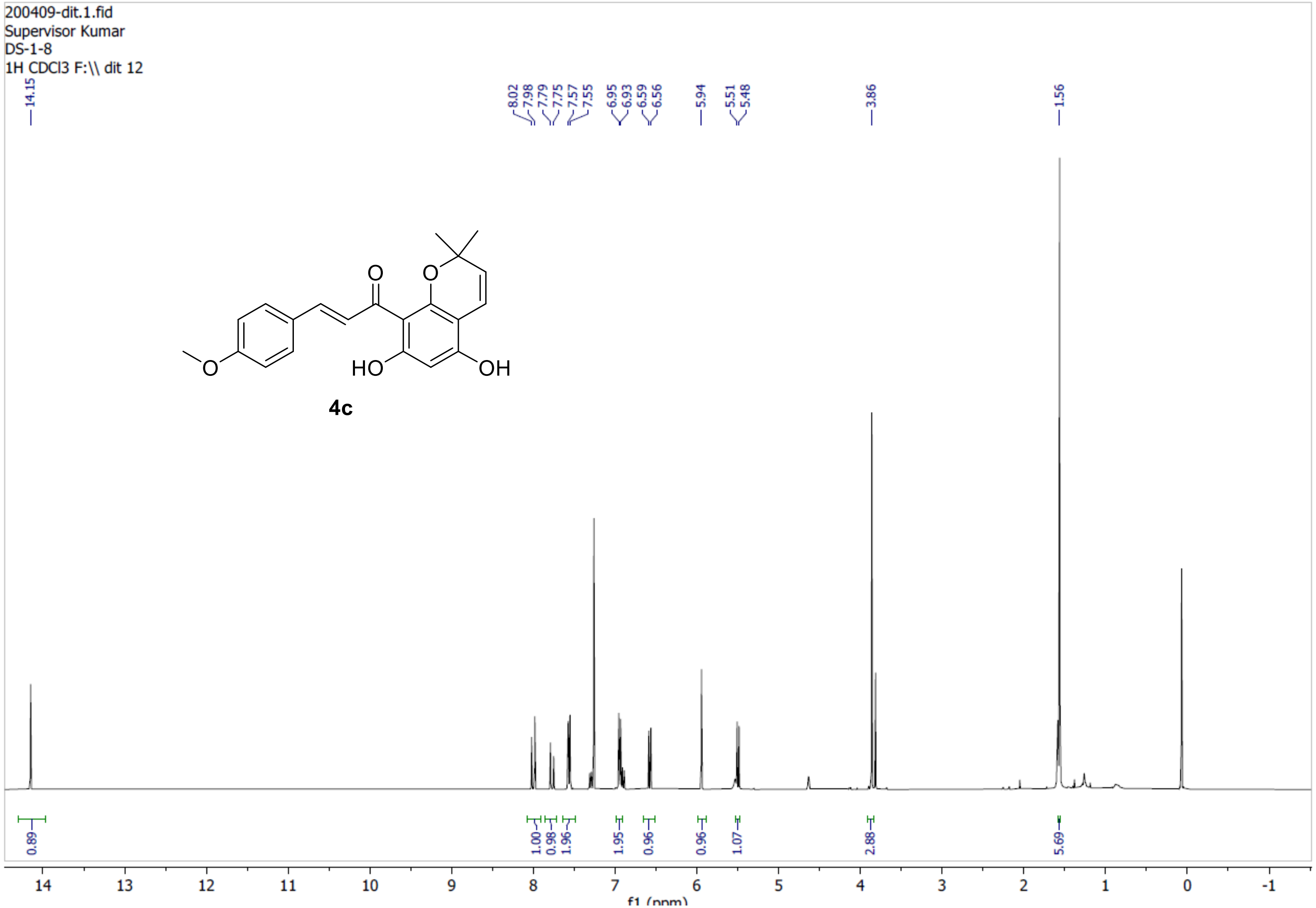

^{1}H NMR spectrum of compound **4d**

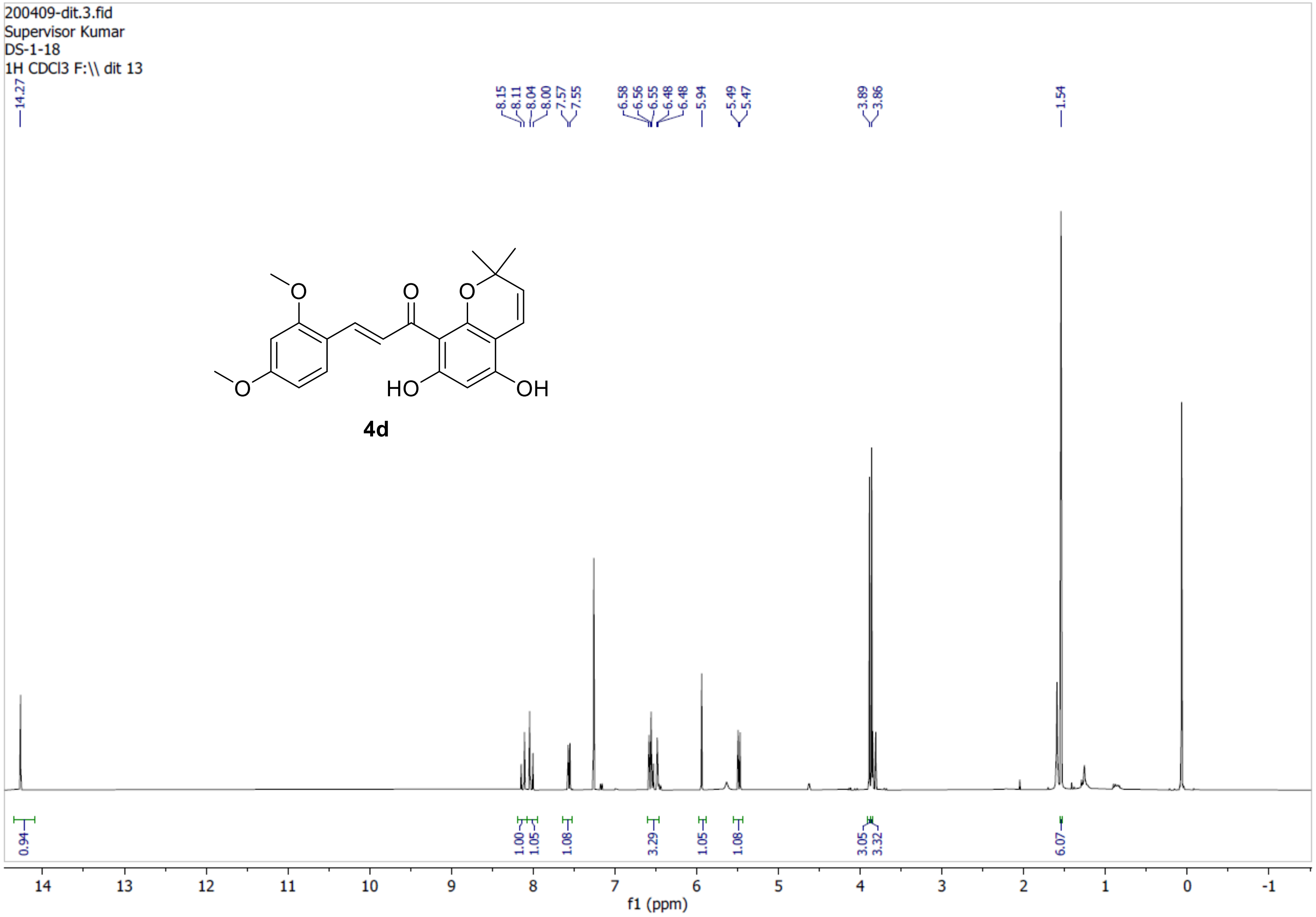

^{13}C NMR spectrum of compound **4d**

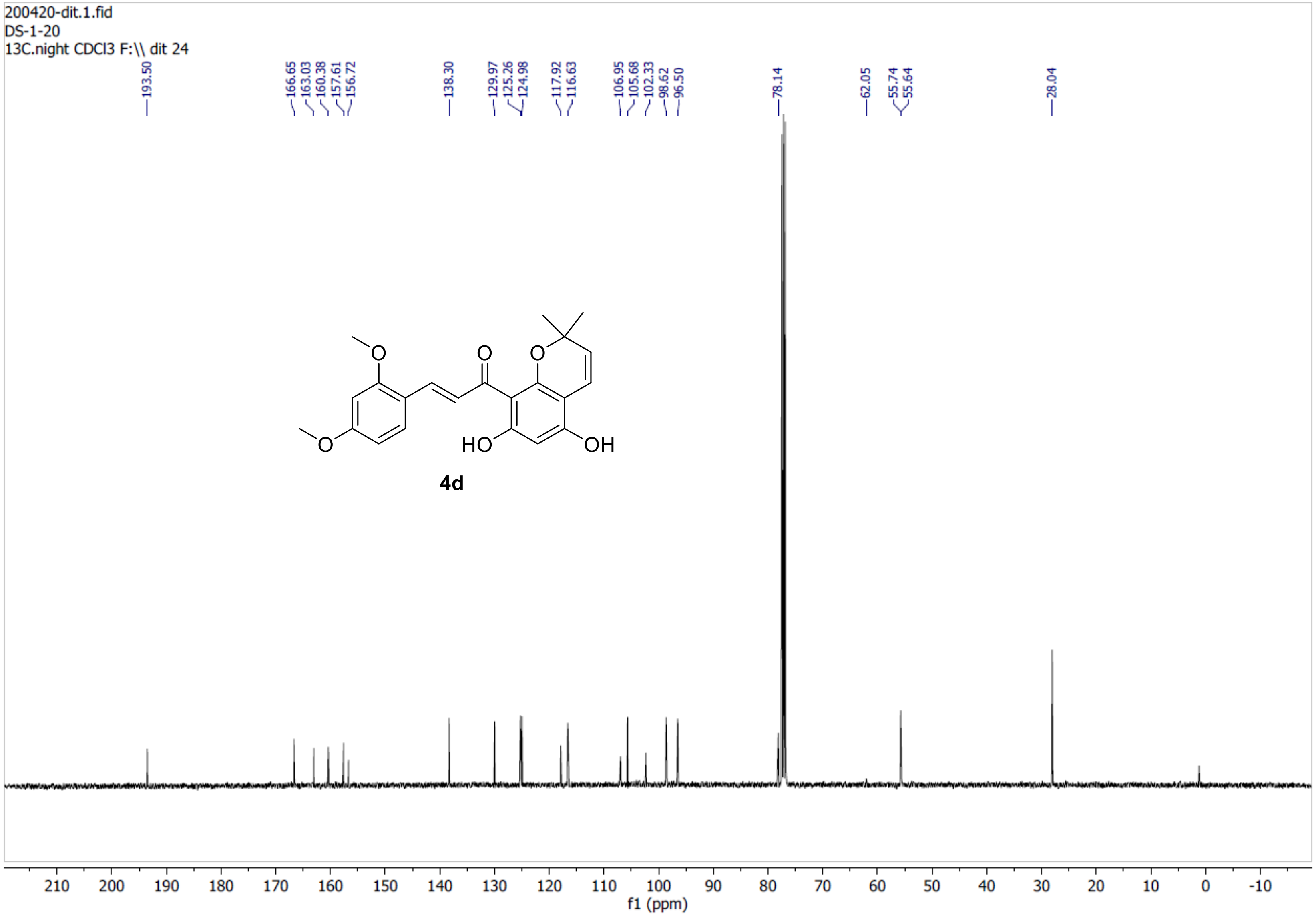

^{1}H NMR spectrum of compound **4e**

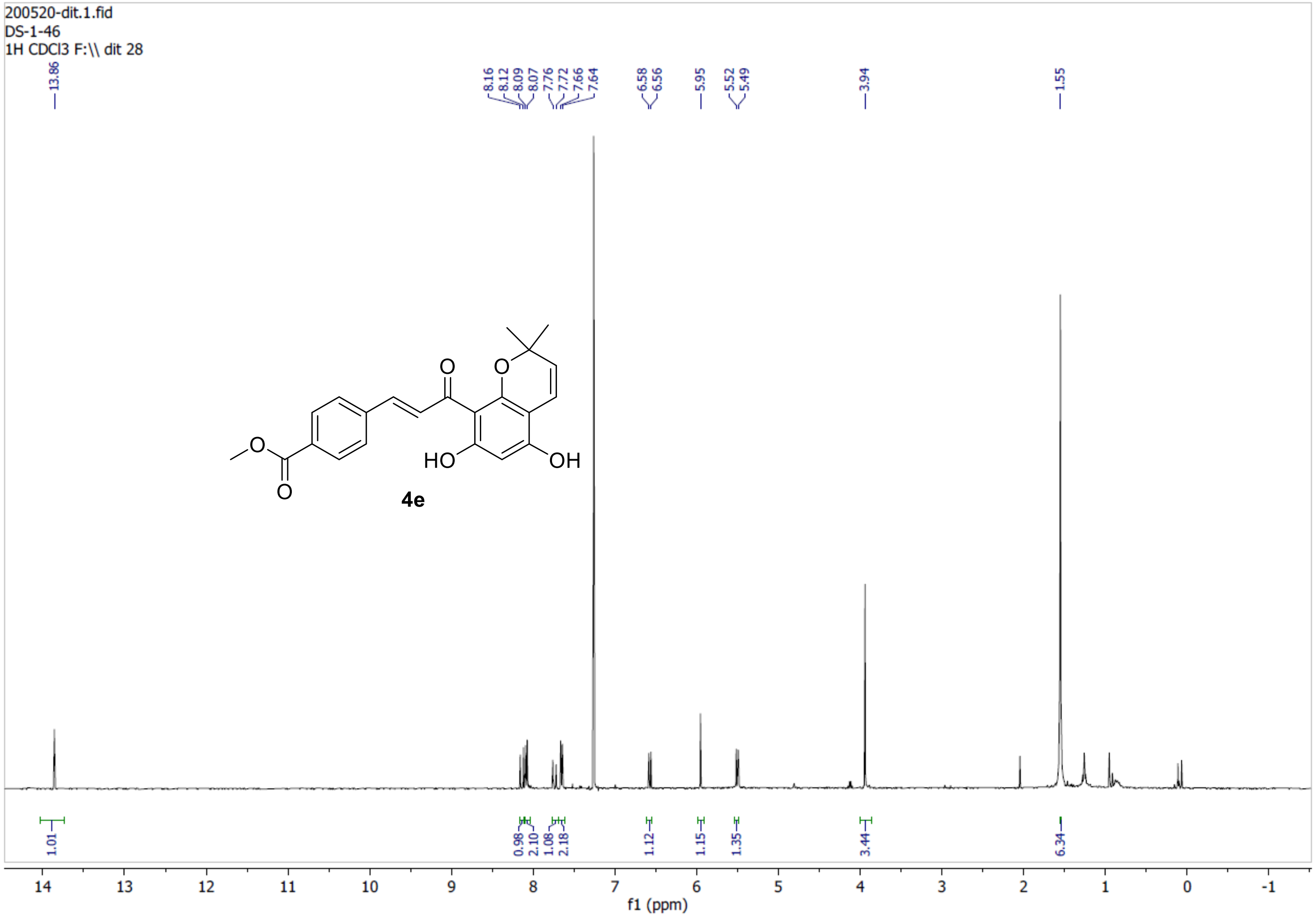

^{1}H NMR spectrum of compound **5a**

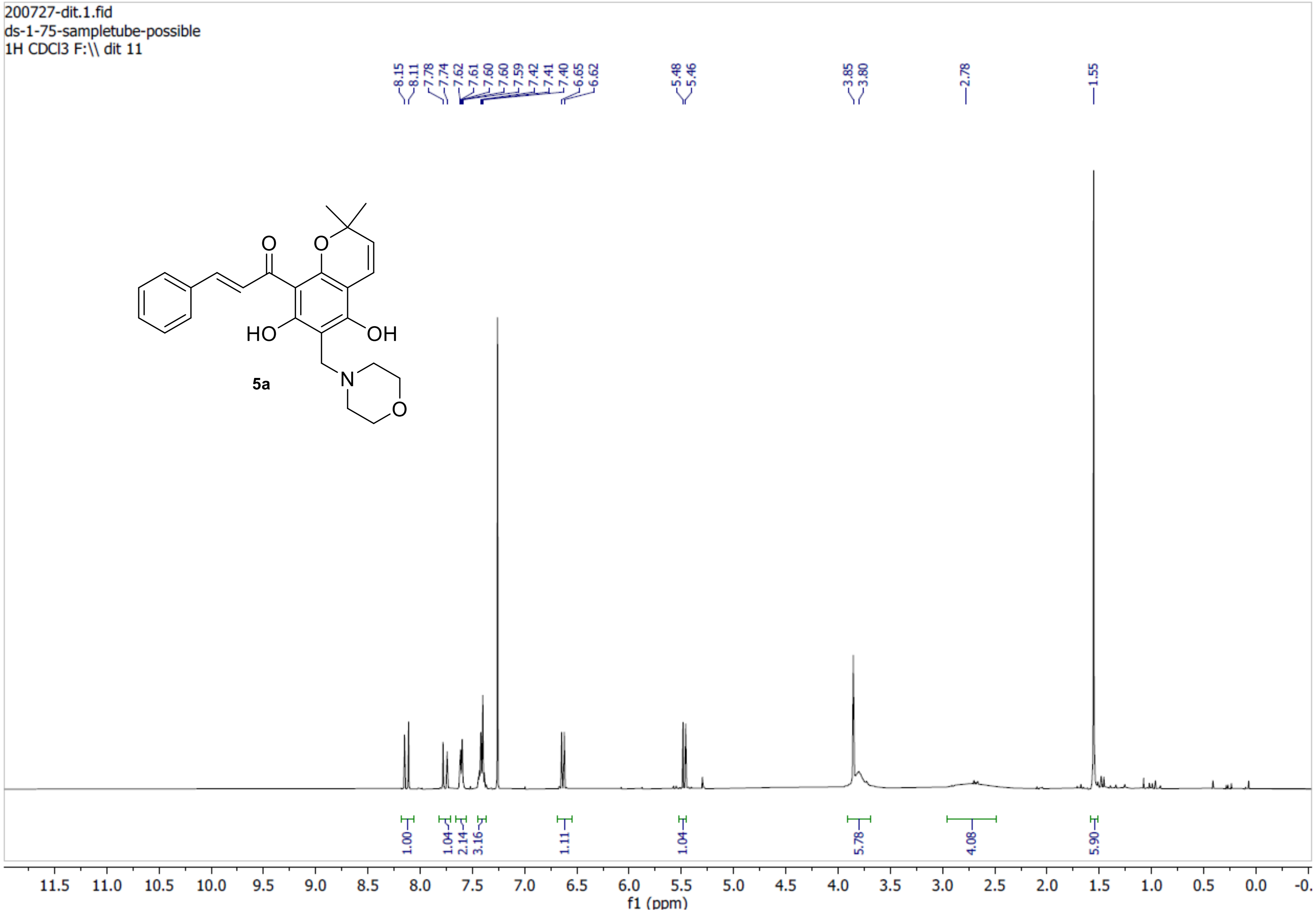

^{13}C NMR spectrum of compound **5a**

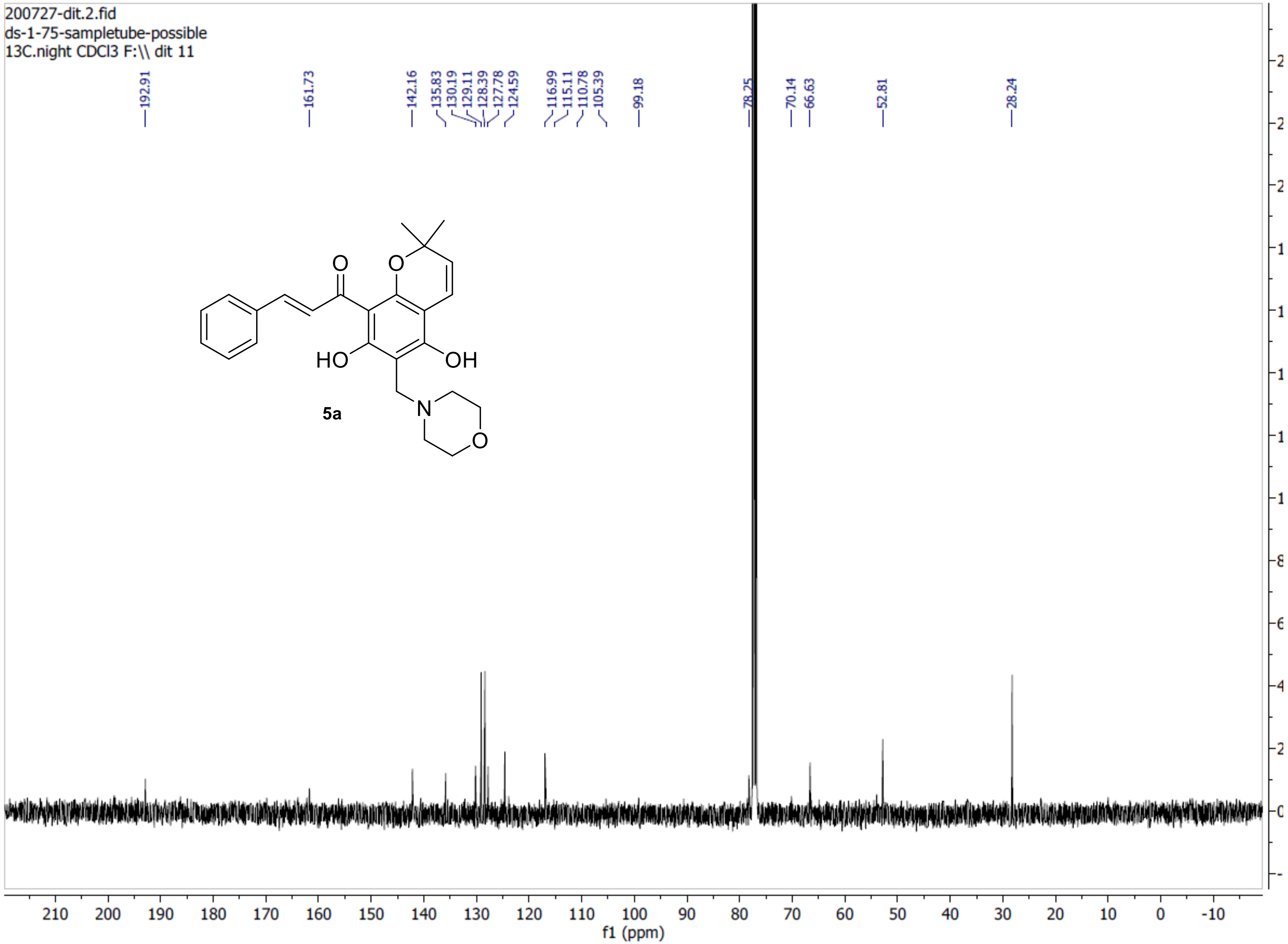

^{1}H NMR spectrum of compound **5b**

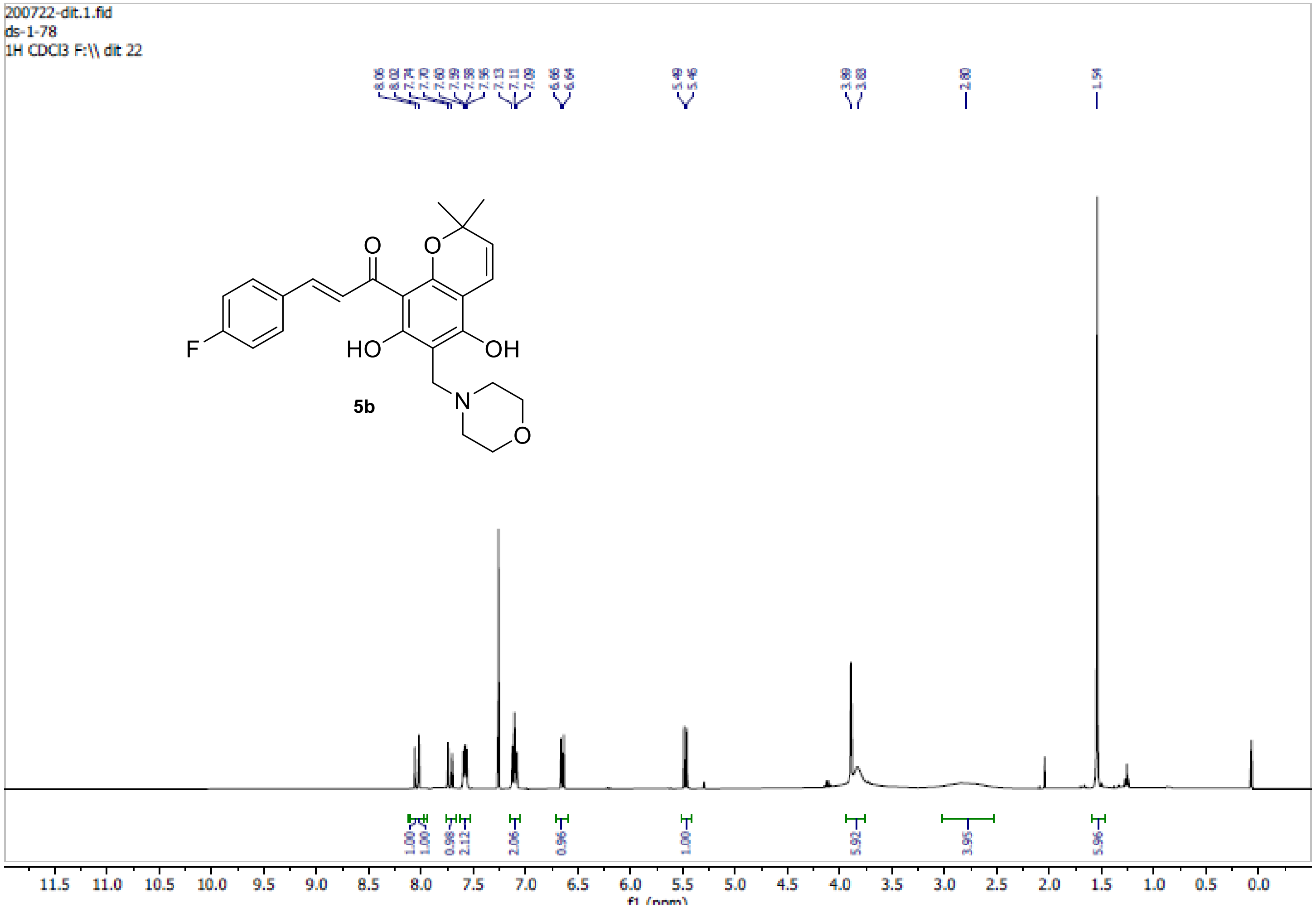

^{13}C NMR spectrum of compound **5b**

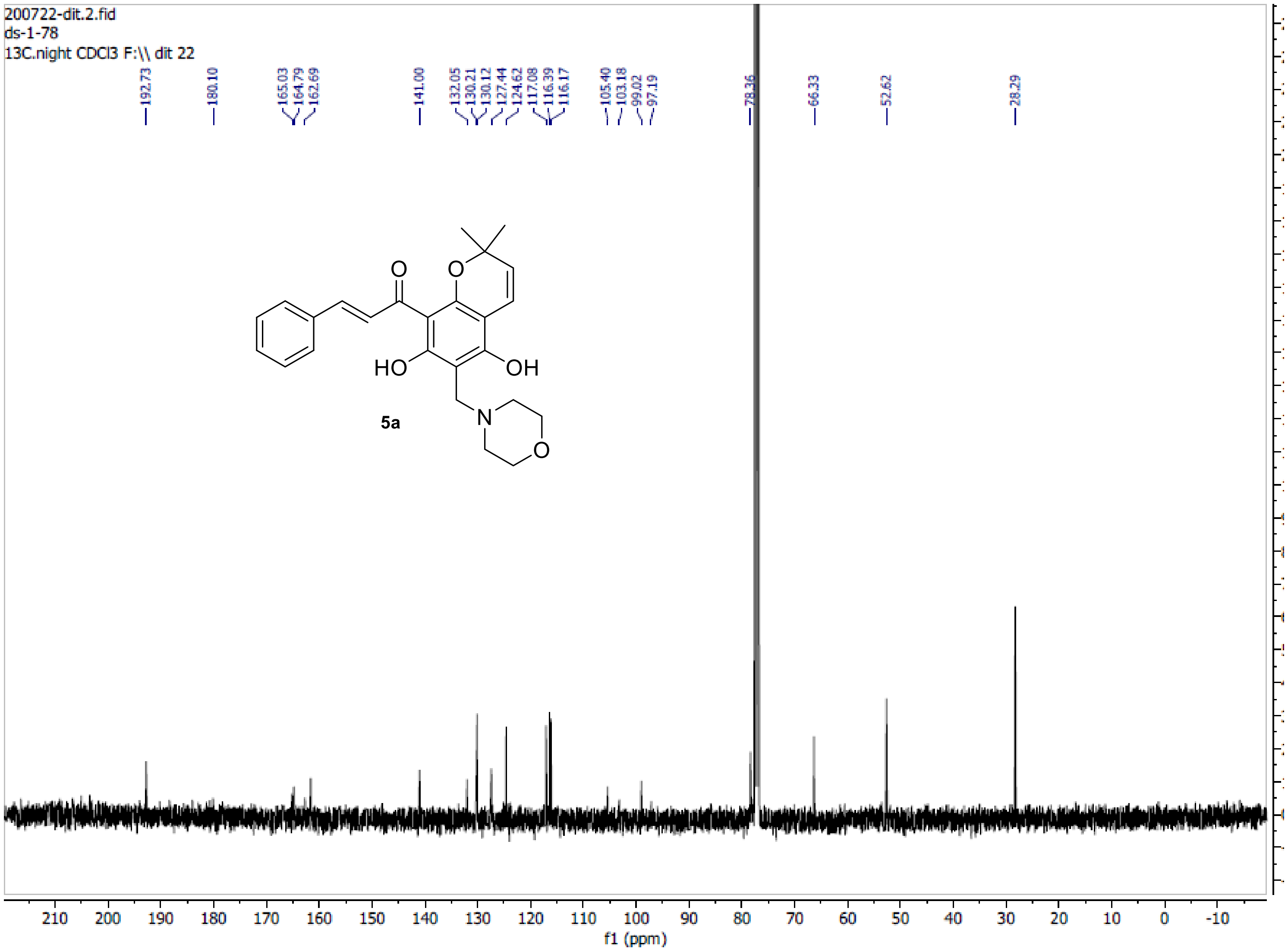

^{1}H NMR spectrum of compound **5c**

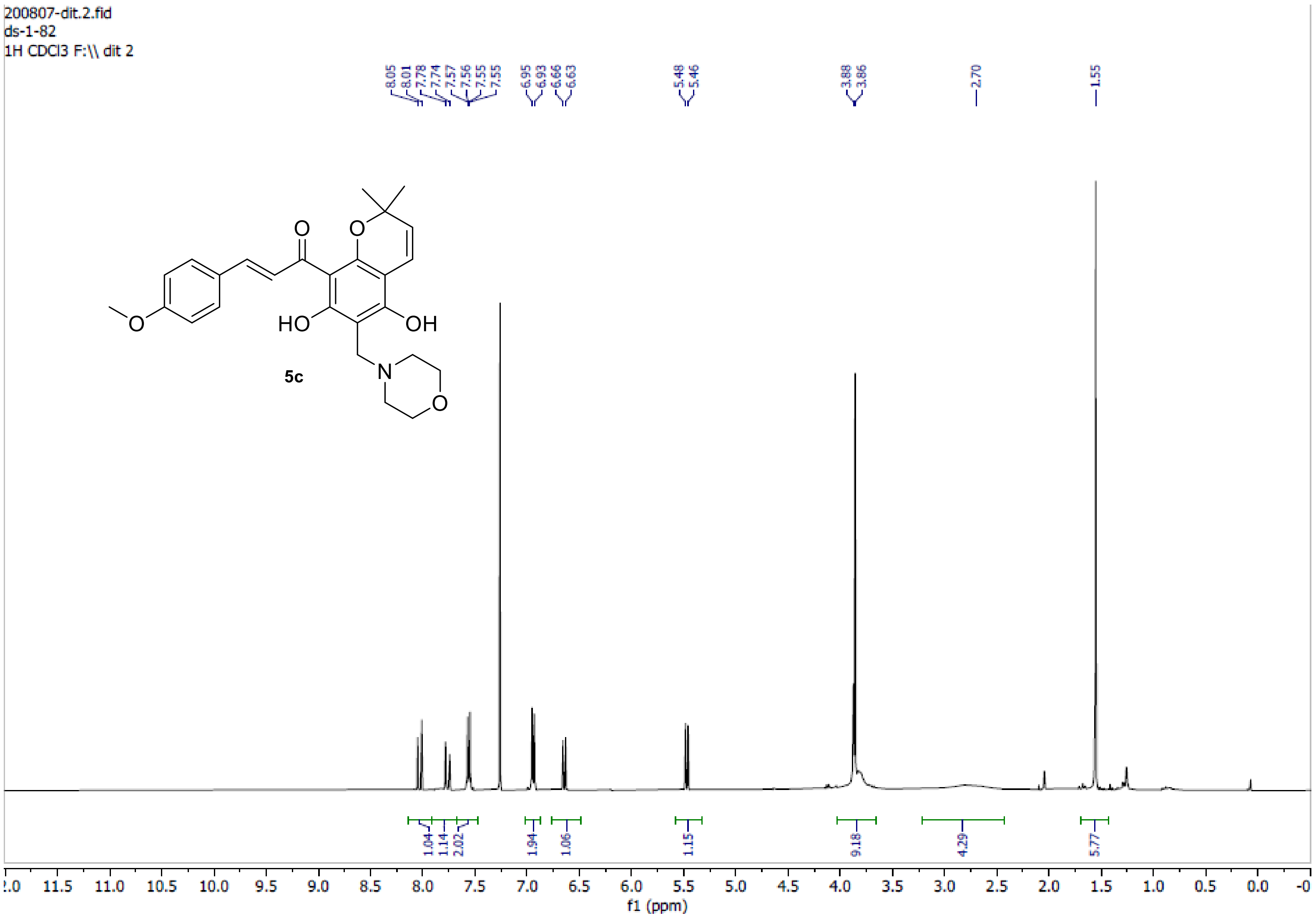

^{13}H NMR spectrum of compound **5d**

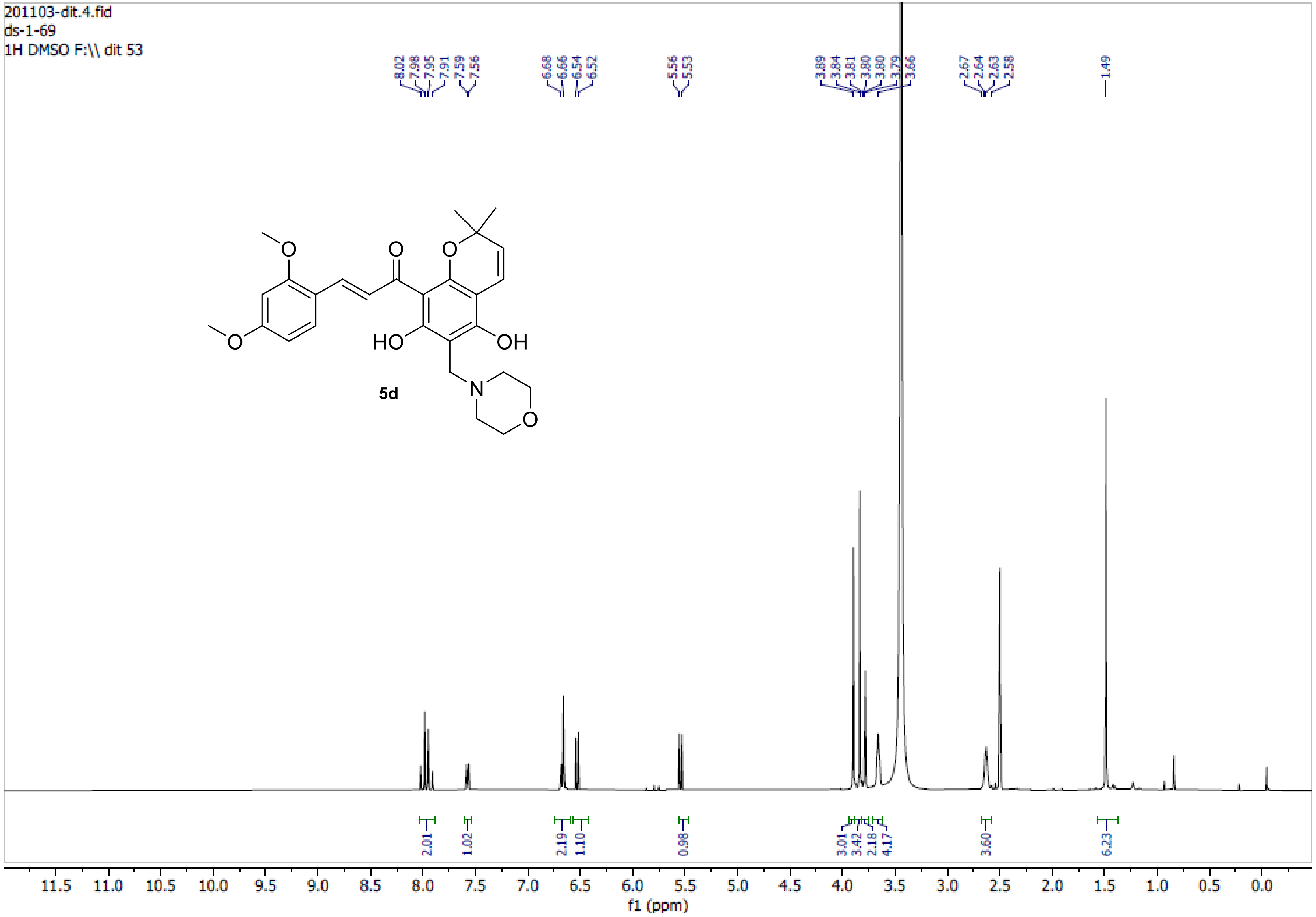

^{13}C NMR spectrum of compound **5d**

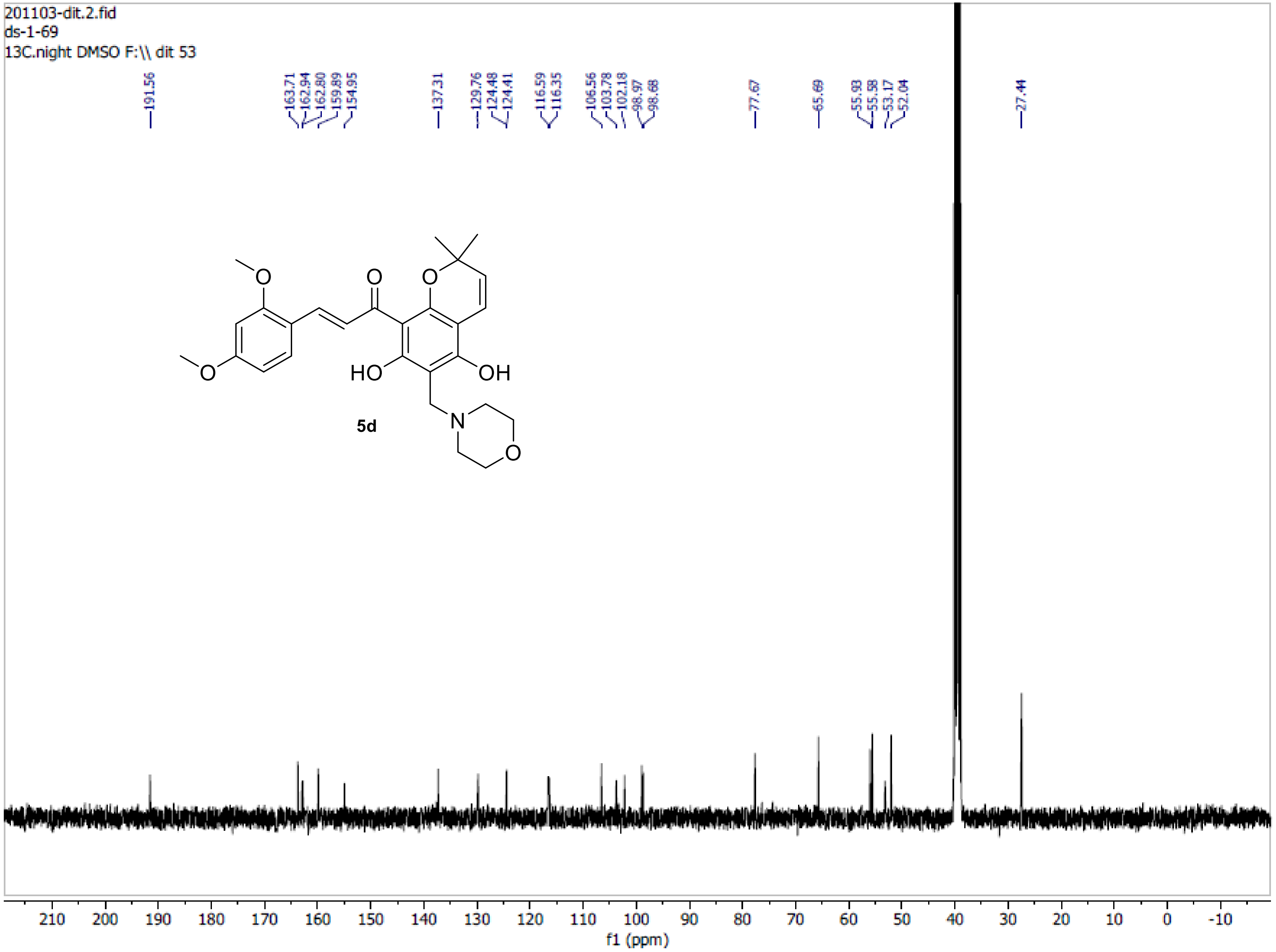

^{1}H NMR spectrum of compound **6**

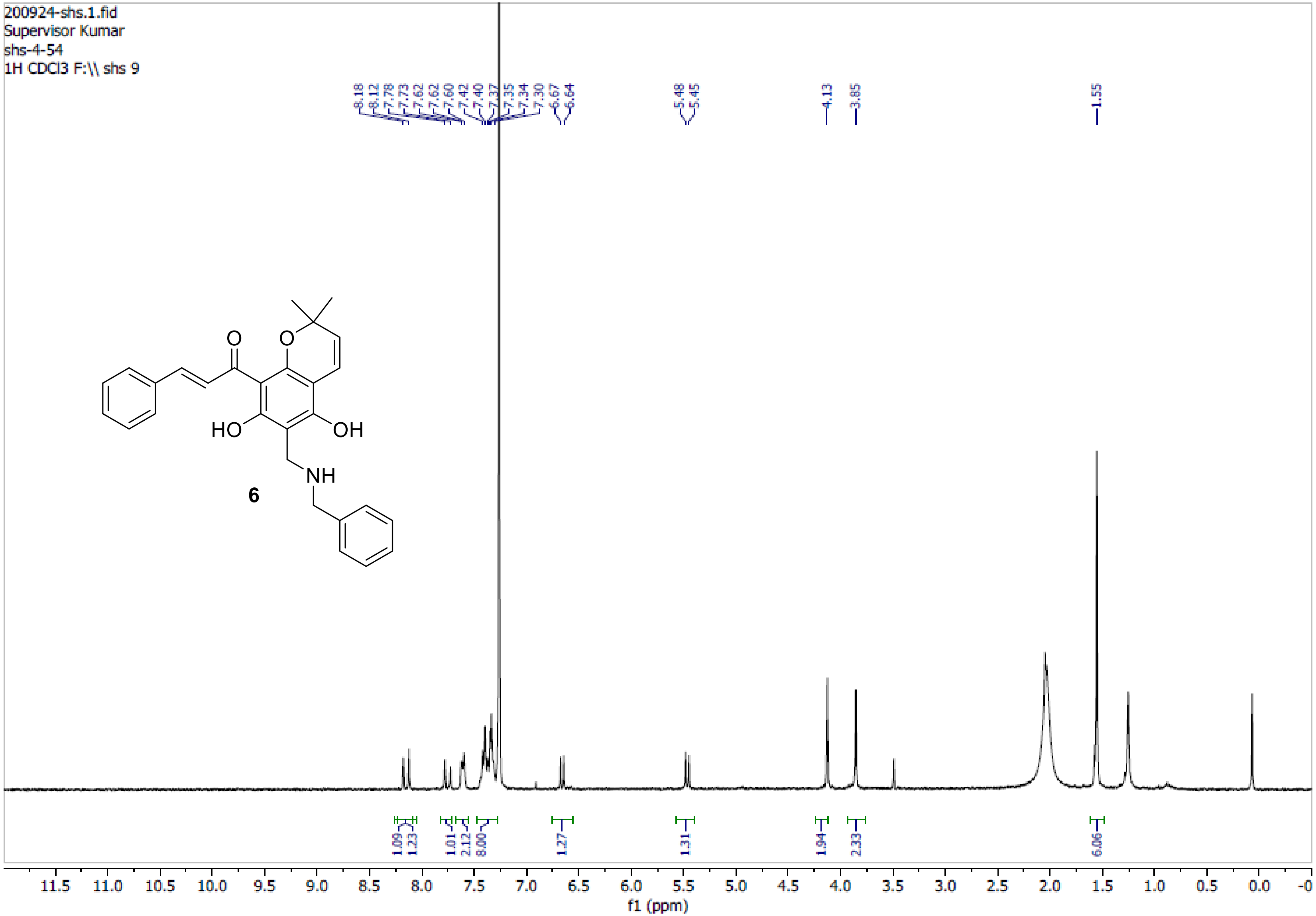

^{13}H NMR spectrum of compound **7**

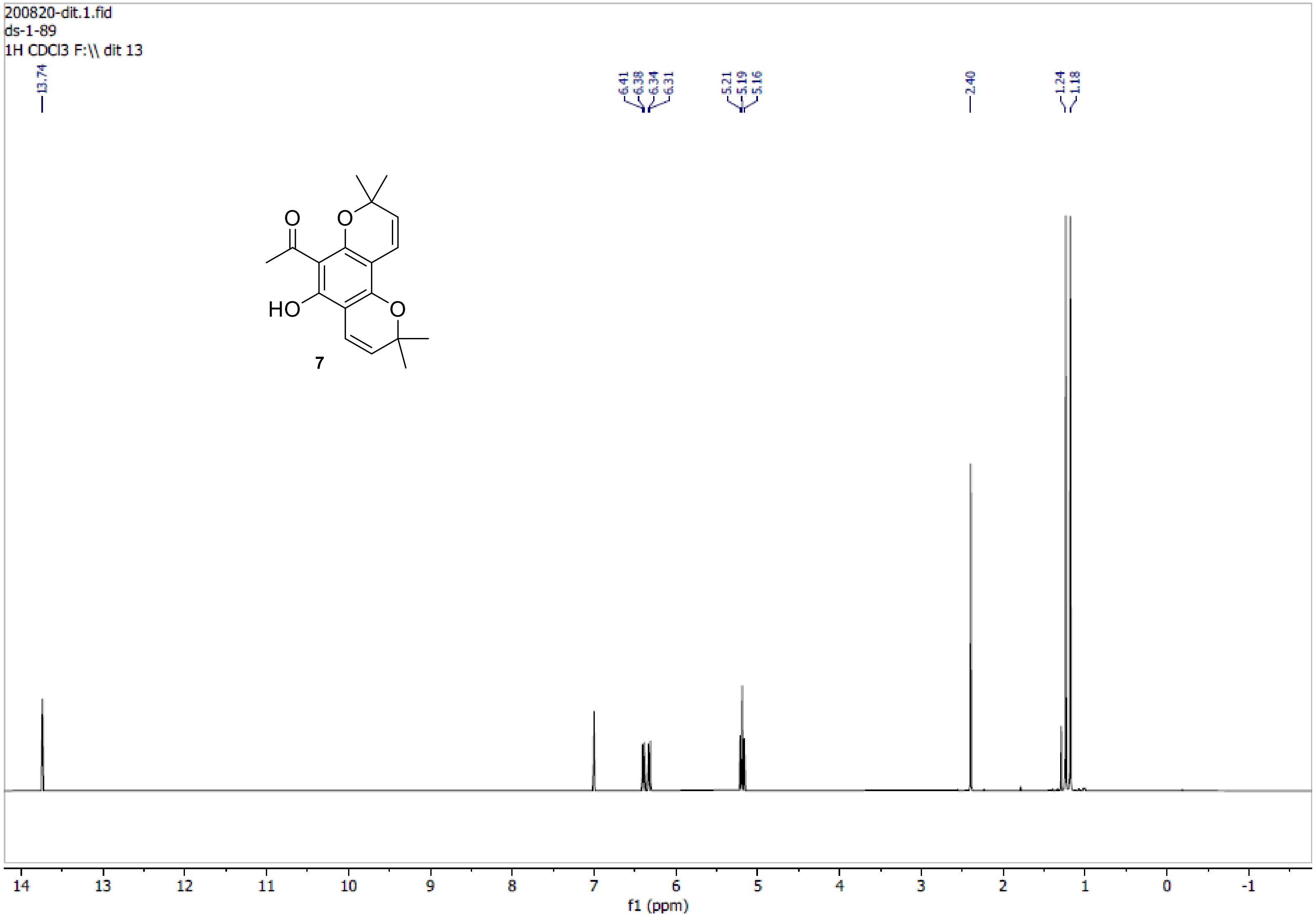

^{13}C NMR spectrum of compound **7**

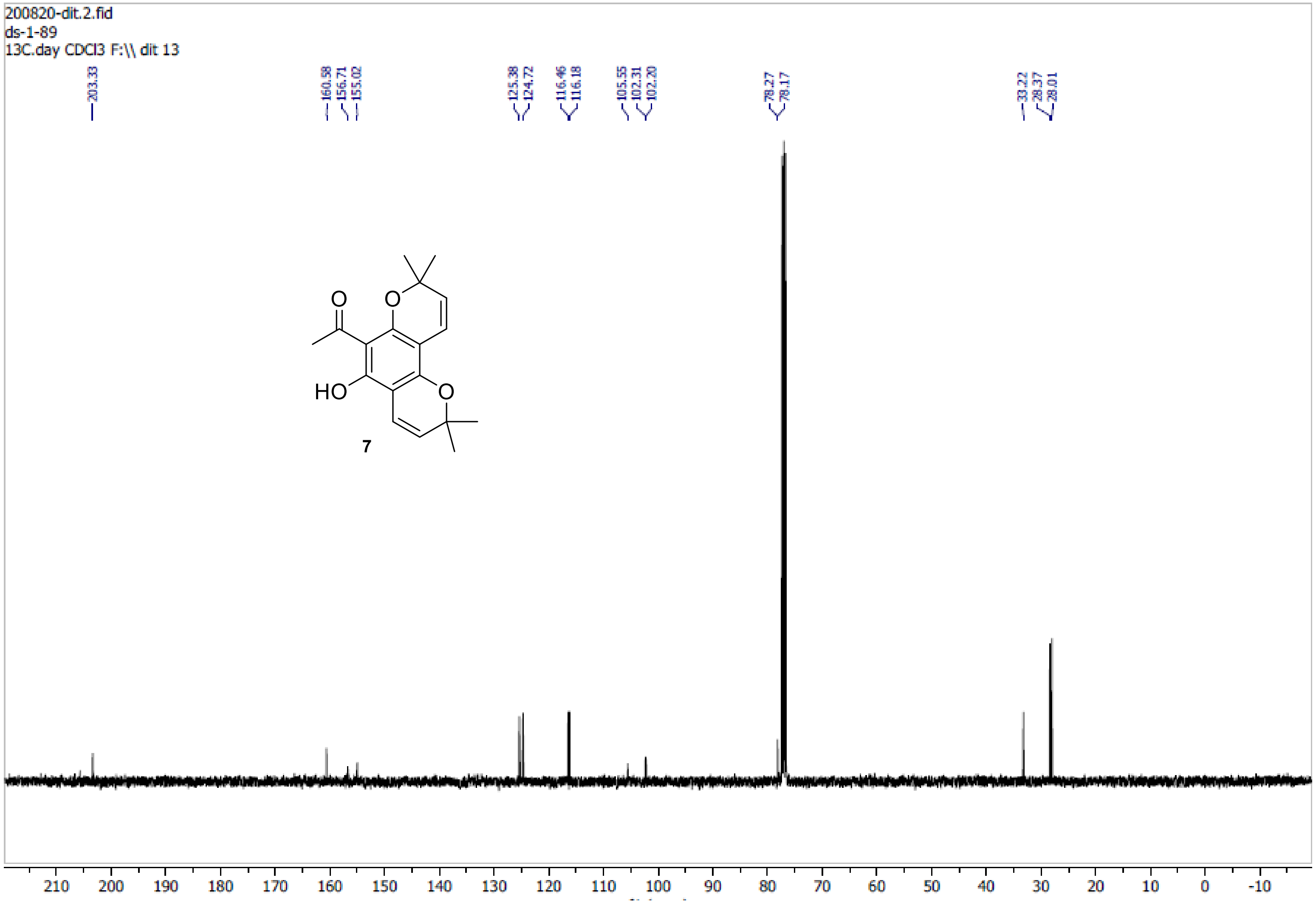

^{1}H NMR spectrum of compound **8a**

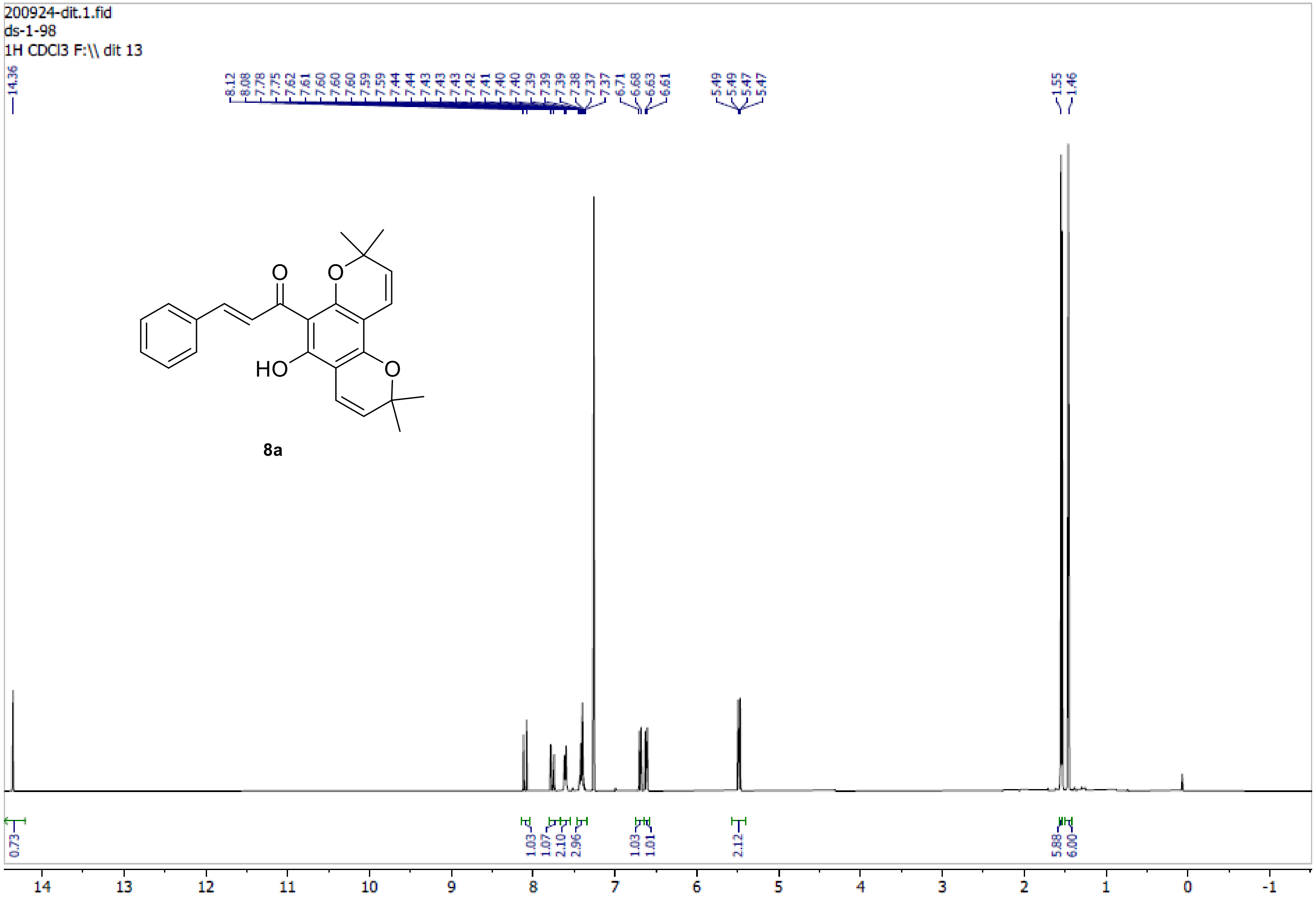

^{13}C NMR spectrum of compound **8a**

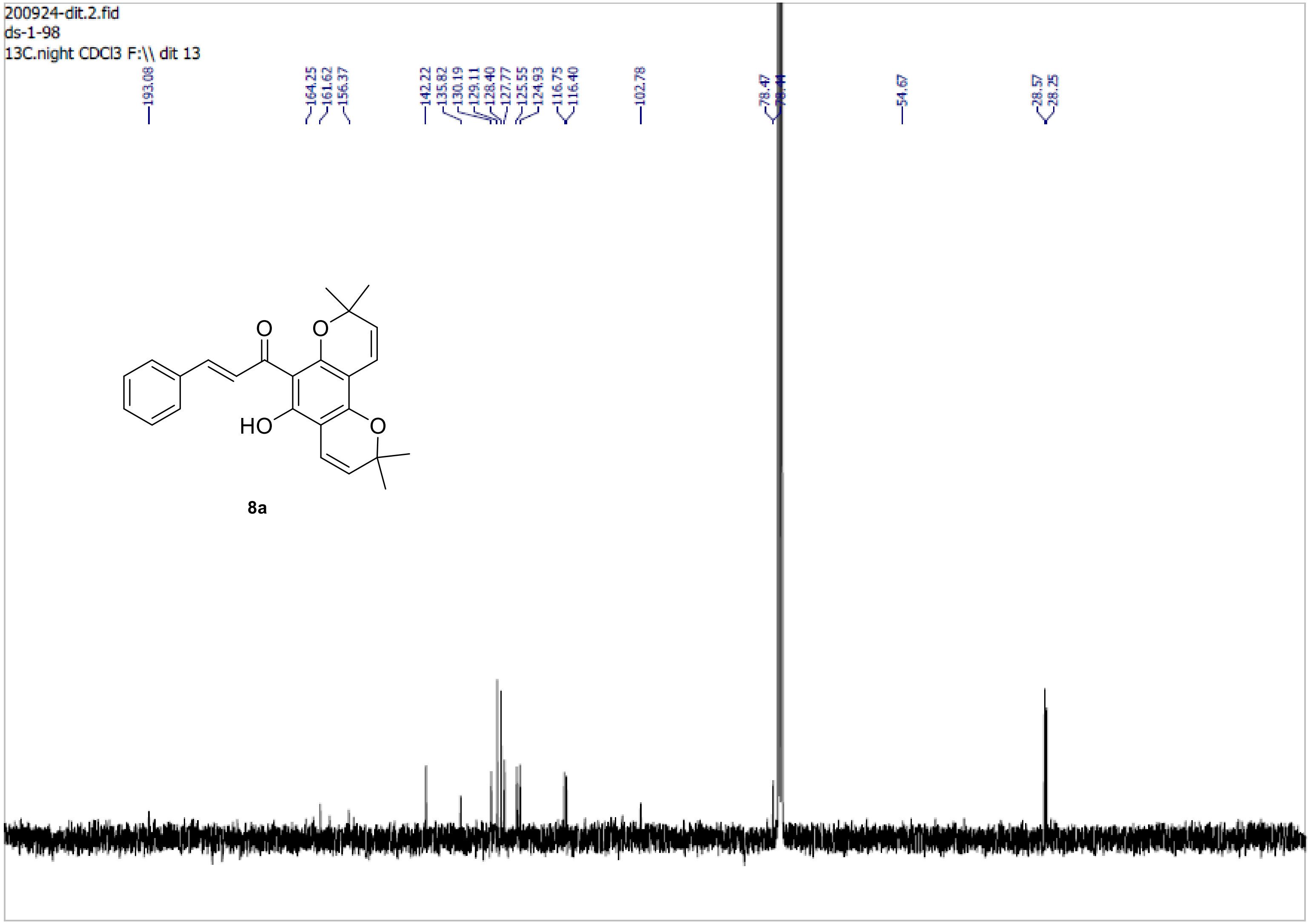

^{1}H NMR spectrum of compound **8b**

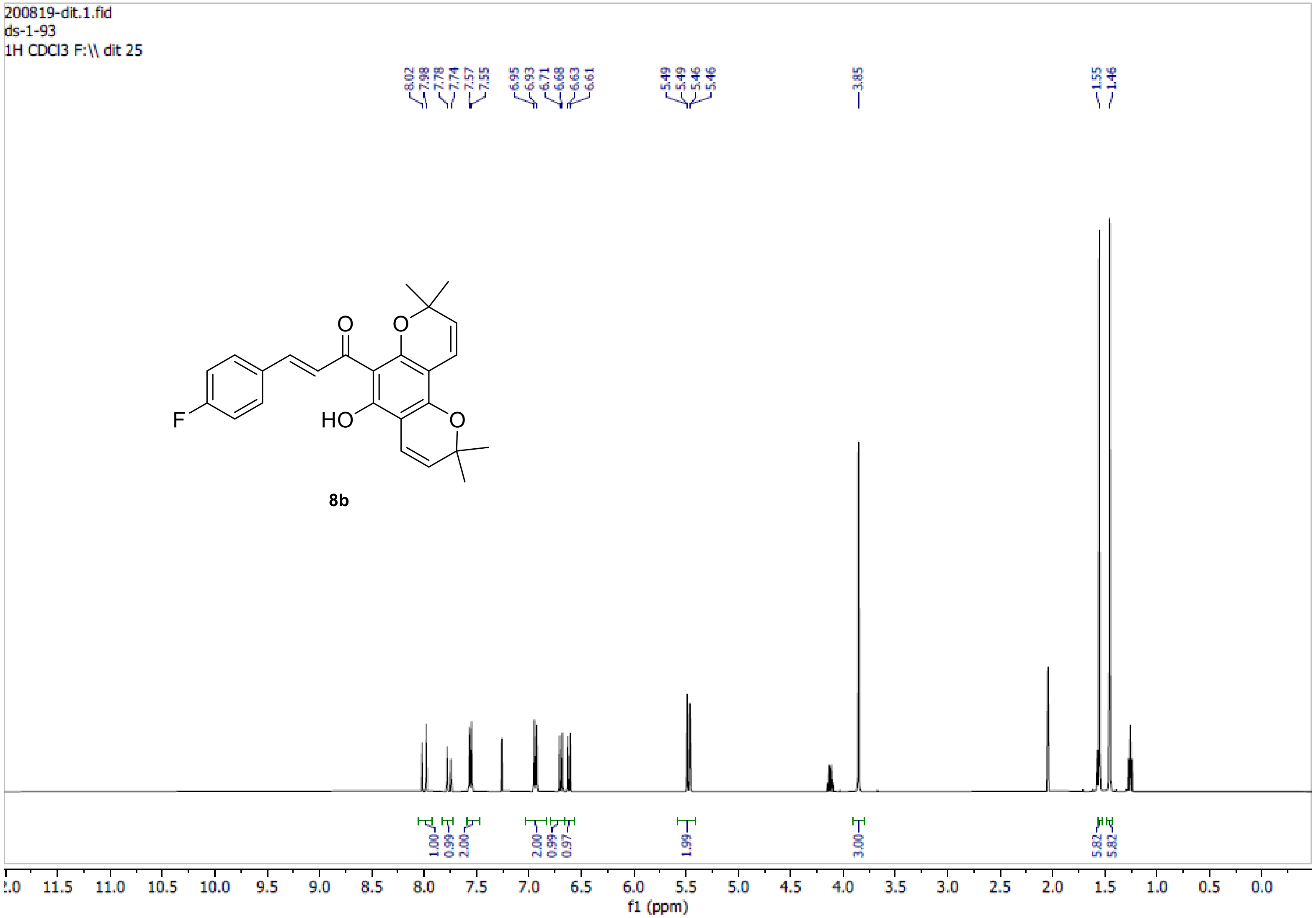

^{1}H NMR spectrum of compound **8c**

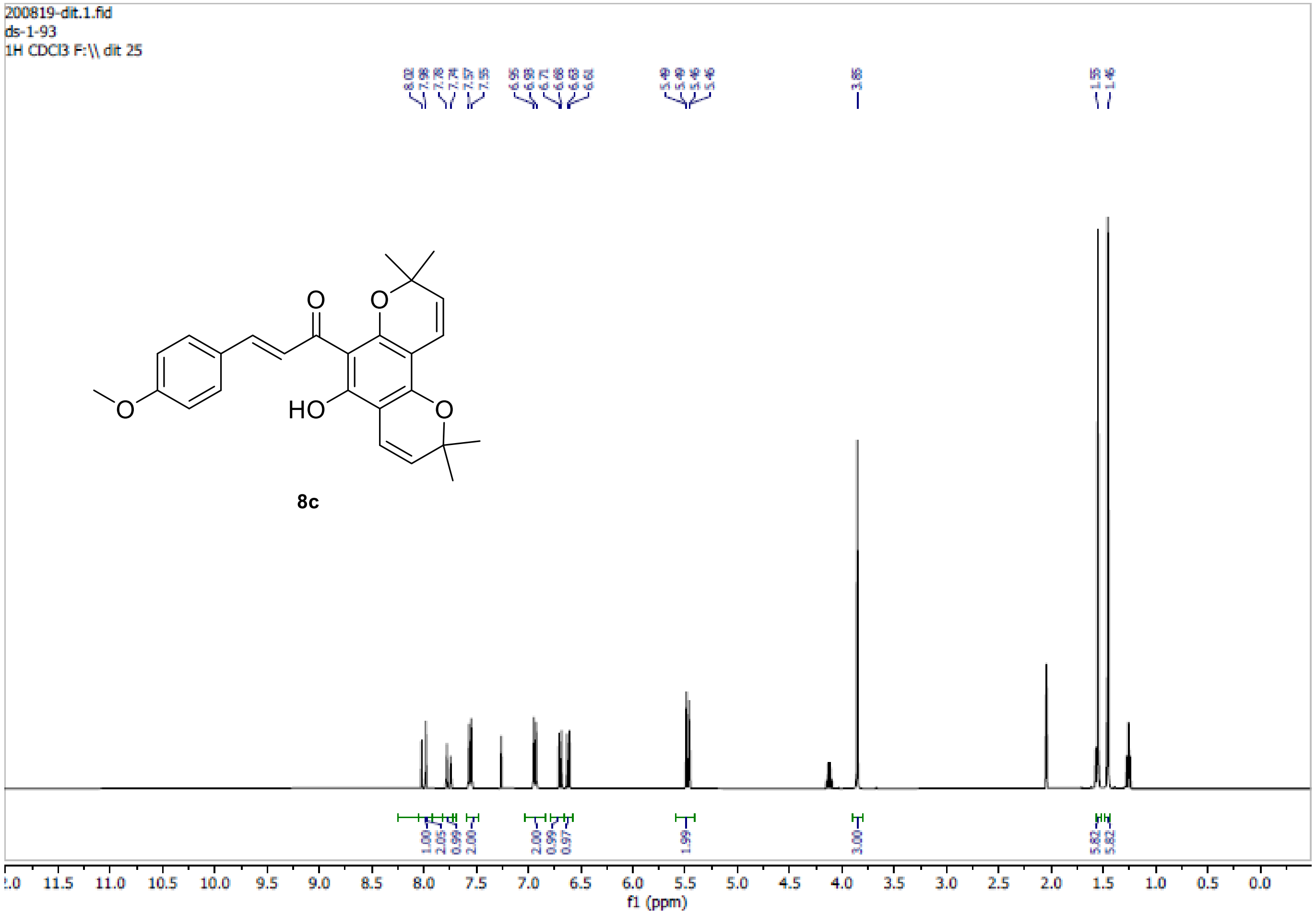

^{13}C NMR spectrum of compound **8c**

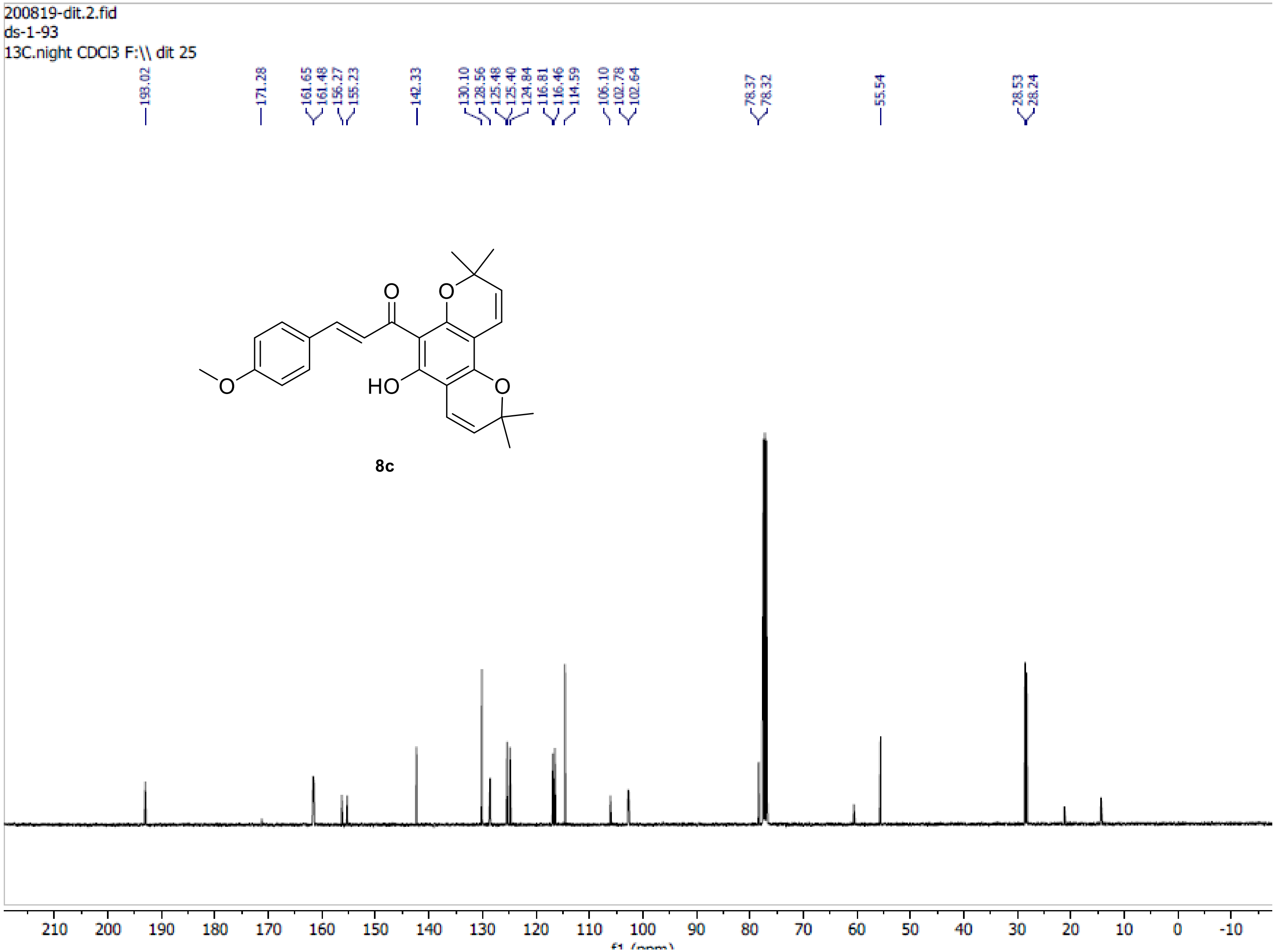

^{1}H NMR spectrum of compound **8d**

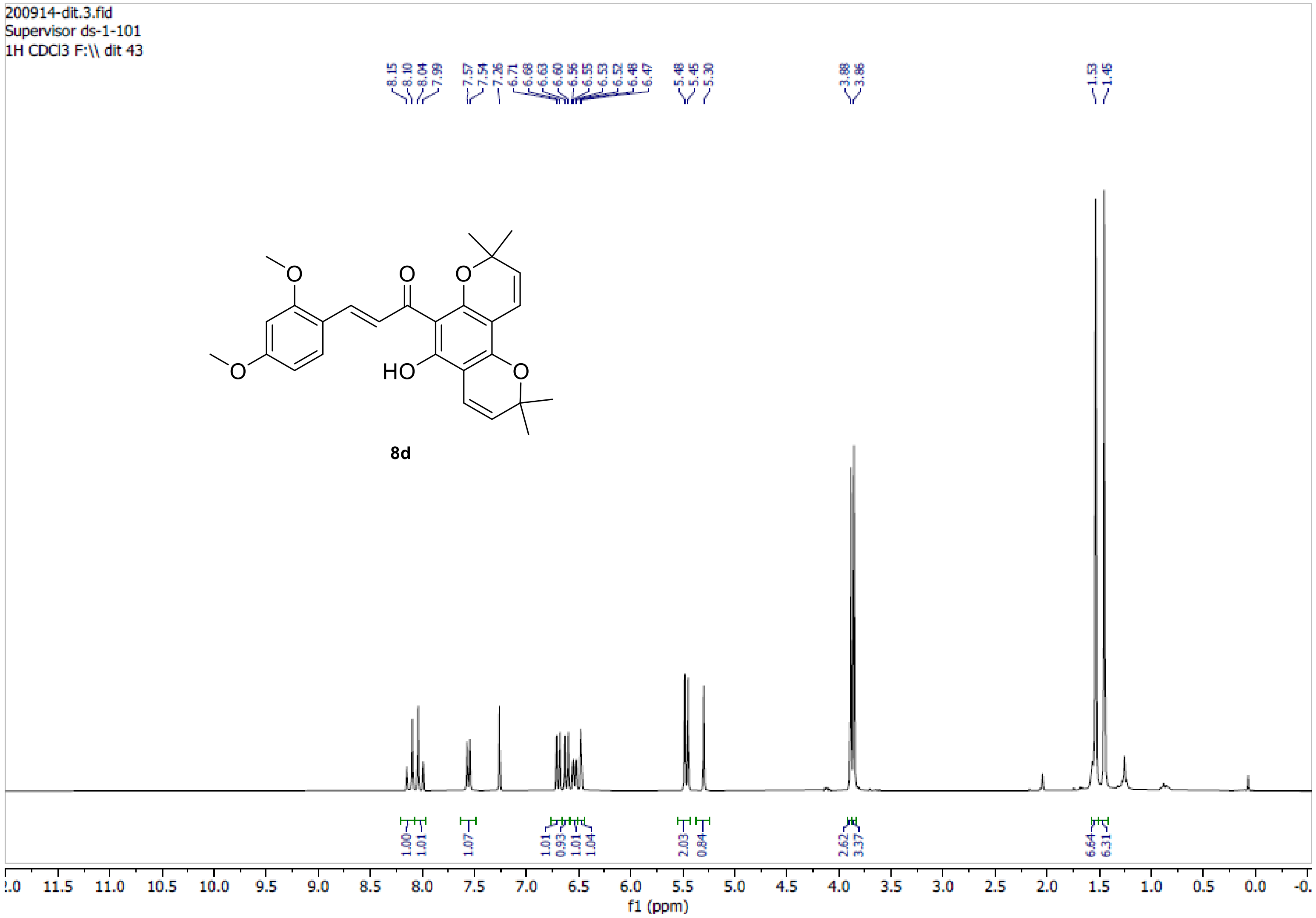

^{13}C NMR spectrum of compound **8d**

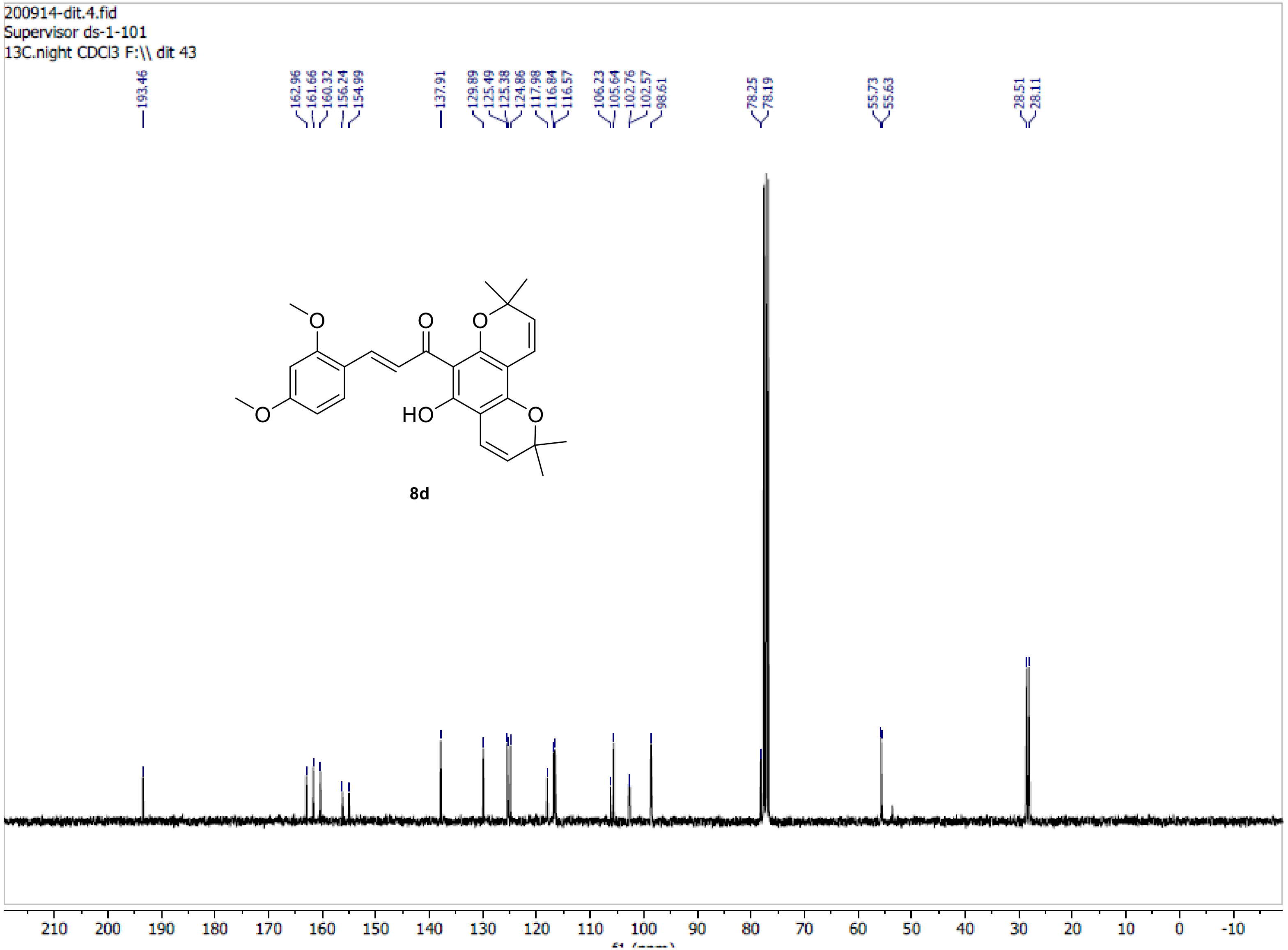

Growth inhibition assay results

The growth of bacteria was measured through observing the OD at λ 600 nm in P_{las}B::*gfp*(ASV). The percentage inhibition of bacterial growth was determined to be the percentage difference in OD value between bacteria treated with compound and control (untreated) bacteria.

Each experiment was performed in triplicate and was repeated in three independent experiments.

**Compound ID**	**125 μM**	**62.5 μM**	**31 μM**
	**Mean percentage inhibition (*P.aeruginosa* MH602)**		
**5a**	19.63 ± 9.66	29.81 ± 9.11	21.18 ± 13.06
**5b**	40.55 ± 4.07	29.39 ± 3.55	30.05 ± 7.37
**5c**	20.80 ± 3.66	4.59 ± 7.3	4.62 ± 3.22
**5d**	12.15 ± 2.81	1.34 ± 2.48	0.73 ± 4.7
**7**	27.51 ± 5.94	13.69 ± 1.02	15.91 ± 6.77
**8a**	7.16 ± 2.31	1.64 ± 3.63	0
**8b**	5.81 ± 7.80	0	0
**8c**	2.62 ± 8.83	3.5 ± 3.8	0
**8d**	1.38 ± 3.36	5.84 ± 8.32	0
